# Profiling of Tregs across tissues reveals plasticity in ST2 expression and hierarchies in tissue-specific phenotypes

**DOI:** 10.1016/j.isci.2022.104998

**Published:** 2022-08-24

**Authors:** Sabine Spath, Florence Roan, Scott R. Presnell, Barbara Höllbacher, Steven F. Ziegler

**Affiliations:** 1 Center for Fundamental Immunology, Benaroya Research Institute, Seattle, WA 98101, USA; 2 Division of Allergy and Infectious Diseases, University of Washington School of Medicine, Seattle, WA 98109, USA; 3 Center for Systems Immunology, Benaroya Research Institute, Seattle, WA 98101, USA; 4 Department of Immunology, University of Washington School of Medicine, Seattle, WA 98109, USA; 5 Institute of Computational Biology (ICB), Helmholtz Zentrum Muenchen (HMGU), 85764 Munich, Neuherberg, Germany; 6 Department of Informatics, TUM, 85748 Munich, Garching, Germany

**Keywords:** Immunology, Components of the immune system, Transcriptomics

## Abstract

Foxp3^+^ regulatory T cells (Tregs) are critical mediators of peripheral tolerance and immune homeostasis and exert tissue-specific functions. In many nonlymphoid tissues, Tregs show enriched expression of the IL-33 receptor ST2. Through comprehensive profiling of murine ST2^+^ and ST2^-^ Tregs, we found that Treg transcriptomes and phenotypes formed a hierarchical relationship across tissues. Only a small core signature distinguished ST2^+^ Tregs from ST2^-^ Tregs across all tissues, and differences in transcriptional profiles were predominantly tissue-specific. We also identified unique, highly proliferative, circulating ST2^+^ Tregs with high migratory potential. In adoptive transfers, both ST2^+^ and ST2^-^ Tregs seeded various host tissues and demonstrated plasticity in ST2 expression. Furthermore, Tregs from donor lungs were differentially recovered from host nonlymphoid tissues in an IL-33-dependent manner. In summary, our work identified tissue residency rather than ST2 expression as a primary driver of tissue Treg identity and highlights the unique, tissue-specific adaption of ST2^+^ Tregs.

## Highlights

• Tissue of residency rather than ST2 expression is a primary driver of Treg identity
• A small core signature distinguishes ST2^+^ Tregs from ST2^-^ Tregs across tissues
• Circulating ST2^+^ Tregs have diverse chemokine receptor profiles
• Plasticity of ST2 expression on transferred Tregs occurs in a tissue-specific manner

## Graphical abstract

**Figure:**
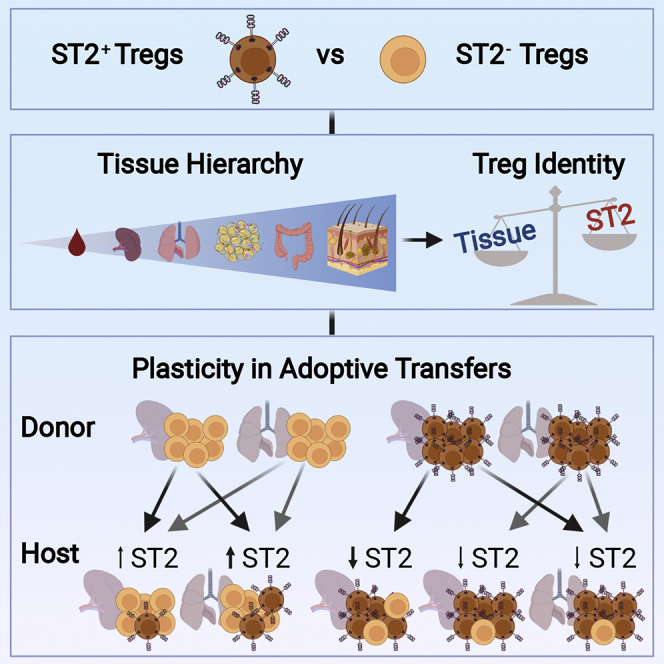

## Introduction

Foxp3^+^ regulatory T cells (Tregs) are a distinct lineage of CD4^+^ T cells required throughout life to maintain self-tolerance (Kim et al., 2007), and the loss or absence of Tregs in humans and mice results in lethal autoimmunity (Bennett et al., 2001; Brunkow et al., 2001; Wildin et al., 2001). A primary function of Tregs is to limit autoimmune-driven inflammation and resolve inflammation after tissue injury or infection. However, Tregs perform a wide breadth of homeostatic functions beyond the suppressive roles that have been classically ascribed to them. Tregs maintain tissue integrity and promote tissue repair after injury at multiple sites including the lungs, skin, heart, skeletal muscle, and CNS (Arpaia et al., 2015; Burzyn et al., 2013; Liesz et al., 2009; Nosbaum et al., 2016; Xia et al., 2020); they help maintain stem cells in the skin, gastrointestinal tract, and bone marrow (Ali et al., 2017; Fujisaki et al., 2011; Hirata et al., 2018); and in the visceral adipose tissue (VAT), they help maintain metabolic homeostasis and regulate insulin sensitivity (Bapat et al., 2015; Feuerer et al., 2009; Kolodin et al., 2015; Vasanthakumar et al., 2015).

Under homeostatic conditions, Tregs are found in both lymphoid and nonlymphoid tissues throughout the body and can be broadly characterized as “resting” central Tregs (cTregs) or “activated” effector Tregs (eTregs) in mice based on the expression of CD44 and CD62L (Smigiel et al., 2014). cTregs are found predominantly in lymphoid tissues and actively recirculate. In contrast, eTregs are enriched in nonlymphoid tissues and are locally maintained to a greater degree (Smigiel et al., 2014), though Treg migration can occur between the skin or colon and the draining lymph nodes even under steady-state conditions (Kolodin et al., 2015; Luo et al., 2016; Nakanishi et al., 2018; Tomura et al., 2010). Some eTreg subpopulations such as those that express CD49b have been shown to be more migratory and perivascular in localization (Fan et al., 2018).

Tregs in nonlymphoid tissues (tissue Tregs) from a variety of sites express the interleukin-33 (IL-33) receptor ST2 (also called IL1RL1) at higher frequencies than their counterparts in lymphoid tissues (Delacher et al., 2017; DiSpirito et al., 2018; Schiering et al., 2014; Vasanthakumar et al., 2015). ST2 has thus emerged as a marker for tissue Tregs. Using a tissue Treg phenotype defined by the expression of KLRG1 and ST2, analyses of chromatin accessibility and single cell transcriptomics identified a Batf-dependent molecular program that can drive differentiation of tissue Treg precursors in lymphoid organs into tissue Tregs (Delacher et al., 2017, 2020). Data from several studies now support a model in which stepwise differentiation and functional specialization of tissue Tregs begins in lymphoid organs but requires local environmental cues within the peripheral tissues for full expression of the tissue Treg phenotype (DiSpirito et al., 2018; Li et al., 2018; Miragaia et al., 2019; Vasanthakumar et al., 2015).

In several tissues, IL-33, the only known ligand for ST2, can drive Treg accumulation. The age-related accumulation of Tregs in adipose tissue and Treg accumulation after tissue injury in skeletal and cardiac muscle are dependent on IL-33 (Kuswanto et al., 2016; Li et al., 2018; Vasanthakumar et al., 2015; Xia et al., 2020). In addition, systemic administration of IL-33 drives the expansion of Tregs (Hemmers et al., 2021; Matta et al., 2014). However, ST2 is expressed on a wide variety of hematopoietic and non-hematopoietic cell types, and IL-33-mediated expansion of Tregs can occur in the absence of Treg-specific ST2 expression (Hemmers et al., 2021). Thus, the degree to which intrinsic ST2 signaling in Tregs drives the tissue-specific imprinting of Tregs or drives the accumulation, retention, activation, and development of Tregs in tissues is not known. In addition, the factors that drive ST2 expression on Tregs and the relationship between ST2^+^ Tregs and ST2^-^ Tregs within individual tissues or across different tissues also remain poorly understood.

In this study, we found that ST2^+^ Tregs displayed a remarkable diversity in frequency and phenotype across tissues. For an unbiased comparison of ST2^+^ and ST2^-^ Tregs within and across tissues, we performed RNA sequencing (RNAseq) of ST2^+^ CD44^hi^ and ST2^-^ CD44^hi^ Tregs from blood, spleen, lungs, VAT, colon, and skin. Across the tissues studied, Treg transcriptomes displayed a hierarchical order that may represent graded levels of activation or differentiation across tissues. ST2^+^ Tregs shared only a small number of differentially expressed genes that universally distinguished them from ST2^-^ Tregs across all the tissues examined but displayed different transcriptional profiles from ST2^-^ Tregs within individual tissues, particularly in the blood. Of note, the frequency of Tregs that expressed ST2 in the blood was higher than in some tissues such as the colon and circulating ST2^+^ Tregs had a diverse chemokine receptor profile suggesting a high migratory potential. Through adoptive transfers, we demonstrate plasticity in Treg ST2 expression, indicating that ST2 expression on Tregs does not represent a terminal activation or differentiation step. In addition, ST2^+^ Tregs from the lungs had a diminished ability to repopulate certain nonlymphoid tissues in IL-33-deficient hosts and thus may have developed a requirement for IL-33 signaling for their accumulation.

## Results

### ST2^+^ Tregs vary in frequency and phenotype across tissues

**Figure 1:**
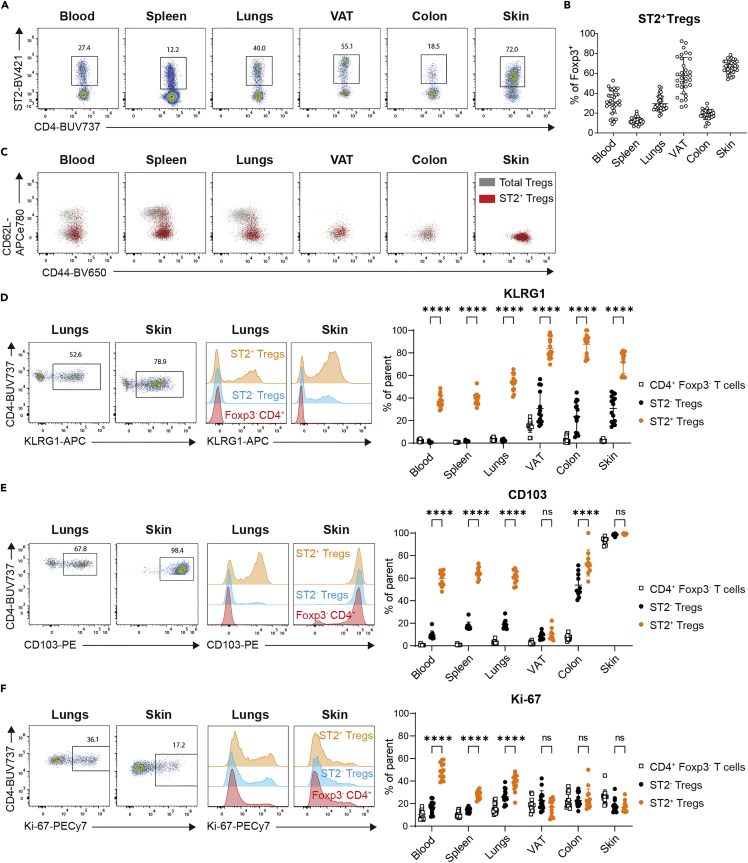
Frequency and phenotype of ST2^+^ Tregs across tissues The frequency and phenotype of Tregs from Foxp3^YFP−cre^ mice were assessed by flow cytometry. (A) Representative flow plots showing ST2 vs CD4, gated on live singlets, CD45^+^, TCRβ^+^ CD4^+^ FOXP3^+^ cells in blood, spleen, lungs, VAT, colon, and skin. (B) Frequency of ST2-expressing Tregs across tissues, based on gating in Figure 1A. Data are pooled from 7 experiments, each with n = 4–6 per cohort. (C) Representative overlays of ST2^+^ Tregs (red) on total Tregs (gray), showing CD62L vs CD44 expression in indicated tissues, pregated on live singlets, CD45^+^, TCRβ^+^ CD4^+^ FOXP3^+^ cells. (D–F) Left: representative flow plots and histograms showing staining of (D) KLRG1, (E) CD103, and (F) Ki-67 in selected tissues and populations (orange: ST2^+^ Tregs, blue: ST2^-^ Tregs, red: Foxp3^-^ CD4^+^ T cells), Right: frequency of (D) KLRG1, (E) CD103, and (F) Ki-67 on ST2^+^ Tregs, ST2^-^ Tregs, and Foxp3^-^ CD4^+^ T cells across tissues. D and F: Data are pooled from 3 experiments of n = 5 each. E: Data are pooled from 2 experiments of n = 5 each, except for VAT and colon with n = 4 in one of 2 experiments. Data are plotted as mean ± SD and indicated means were compared with two-way ANOVA with Sidak’s multiple comparisons test, ns p > 0.05, ∗∗∗∗p < 0.0001. Abbreviations: VAT: visceral adipose tissue, TCR: T cell receptor. See Figure S1 for flow cytometric gating and analysis of Tregs across tissues and in secondary lymphoid organs.

Tregs in nonlymphoid tissues are functionally and phenotypically distinct from those in lymphoid organs. Several studies have identified the IL-33 receptor ST2 as a marker of tissue Tregs (Delacher et al., 2017; DiSpirito et al., 2018; Schiering et al., 2014; Vasanthakumar et al., 2015). However, the extent to which tissue residency drives ST2 expression on Tregs and whether the phenotype of ST2^+^ Tregs differs in different tissue microenvironments is not well known. To address this open question, we performed an extensive analysis of Tregs from blood, spleen, lungs, abdominal VAT, colonic lamina propria, and back skin in mice. Within the tissues analyzed, the frequency of Tregs among CD4^+^ T cells was lowest in blood, spleen, and lungs and highest in VAT, colon, and skin (Figures S1A and S1B). The frequency of Tregs expressing ST2 was lowest in spleen and highest in VAT and skin (Figures 1A and 1B). Given that ST2 is considered a marker for tissue Tregs, we were surprised to find that about 30% of Tregs in the blood expressed ST2, which was approximately equivalent to Tregs in the lungs and higher than Tregs in the colon (Figures 1A and 1B). Since sex-specific differences have been reported for Tregs in the VAT (Vasanthakumar et al., 2020), we also analyzed males and females separately. The frequency of both Tregs and ST2^+^ Tregs in VAT was lower in females than in males, whereas no sex-specific differences were found in blood, spleen, lungs, colon, or skin (Figures S1C and S1D). Within lymph nodes (LN) draining the lungs, colon, or skin, Treg and ST2^+^ Treg frequencies were comparable to frequencies found in the spleen (Figures S1E and S1F).

Within peripheral nonlymphoid tissues, most Tregs are eTregs, a subset that has been commonly defined by high expression of CD44. We found that only CD44^hi^ Tregs expressed ST2 (Figure 1C), suggesting that ST2 may be expressed exclusively by eTregs. In tissues like blood, spleen, and lungs that harbor a population of CD62L^+^ Tregs, a small subset of ST2^+^ CD44^hi^ Tregs co-expressed CD62L (Figure 1C). Across all tissues, a higher frequency of ST2^+^ Tregs than ST2^-^ Tregs expressed the activation marker KLRG1 (Figure 1D), whereas another eTreg-associated marker CD103 was differentially expressed between ST2^+^ Tregs and ST2^-^ Tregs in blood, spleen, lungs, and colon but not in VAT and skin (Figure 1E). When we assessed proliferation based on the expression of the intranuclear marker Ki-67, we found that a higher frequency of ST2^+^ Tregs than ST2^-^ Tregs expressed Ki-67 in the blood, spleen, and lungs. In VAT, colon, and skin, the frequency of Tregs expressing Ki-67 was low overall but comparable between both Treg populations (Figure 1F). Of all the populations analyzed, ST2^+^ Tregs in the blood had the highest frequency of Ki-67. Across the LNs analyzed, there were small differences in the frequency of ST2^+^ Tregs expressing markers such as CD103, suggesting some contribution of the draining tissues to the LN Treg phenotype; however, a higher frequency of ST2^+^ Tregs than ST2^-^ Tregs expressed KLRG1, CD103, and Ki-67 at all LN sites examined, which also paralleled what was seen in the spleen (Figures S1G–S1I). These data indicate that although ST2 is expressed specifically on activated Tregs, the correlation between ST2 and other markers of Treg activation and differentiation is marker- and tissue specific.

**Figure 2:**
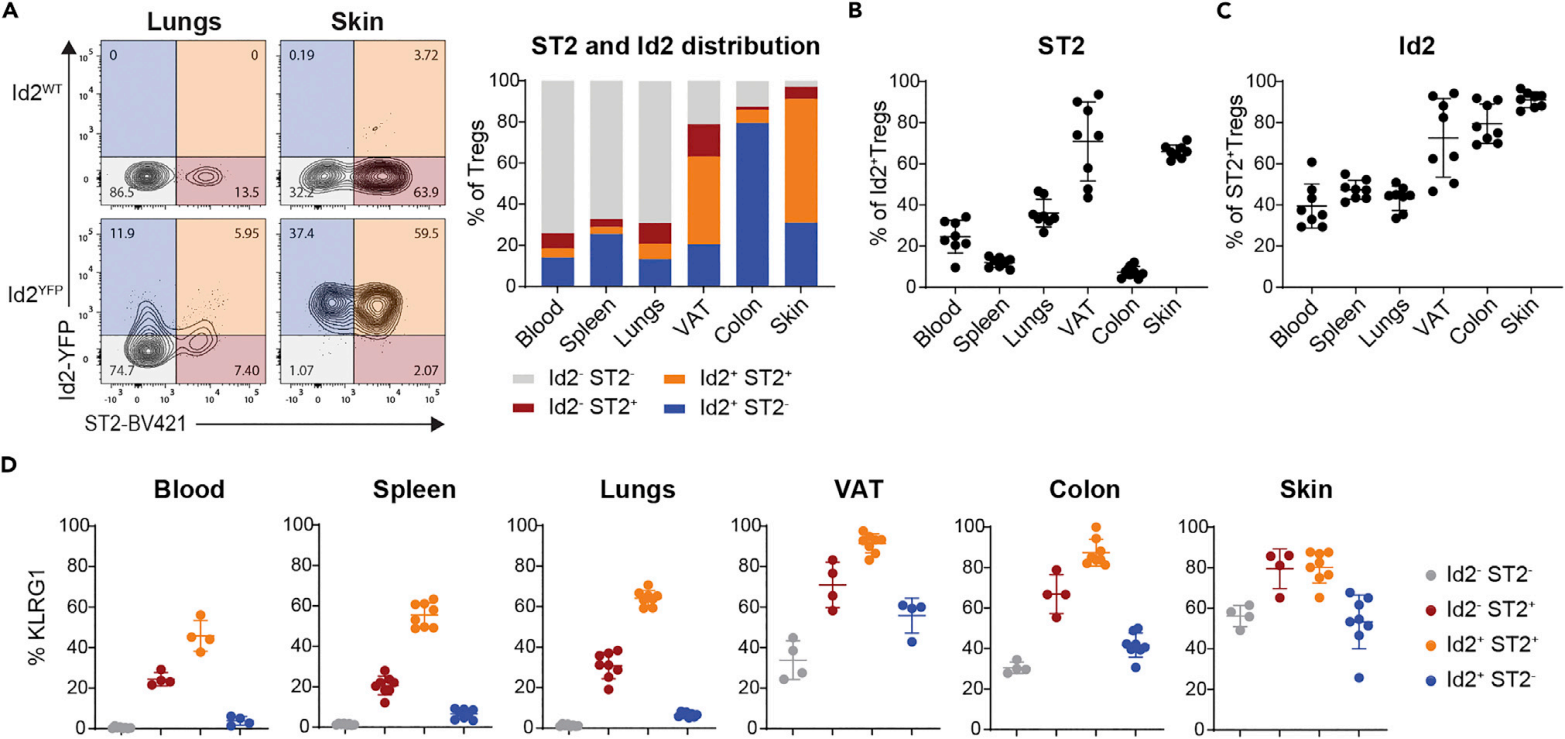
Correlation between Id2^+^ Tregs and ST2^+^ Tregs across tissues The frequency of Id2^+^ and ST2^+^ Tregs from Foxp3^mRFP^Id2^YFP^ reporter mice was assessed by flow cytometry. (A) Relative distribution of Id2 and ST2 on Foxp3^+^ Tregs in indicated tissues, left: representative flow plots, right: quantification. (B) Frequency of ST2^+^ cells among Id2^+^ Tregs in indicated tissues. (C) Frequency of Id2^+^ cells among ST2^+^ Tregs in indicated tissues. (D) Frequency of KLRG1 on indicated Treg populations from Figure 2A in blood, spleen, lungs, VAT, colon, and skin. A–D: Data are pooled from two independent experiments with n = 4 each; for D: data points were removed for populations that routinely had less than 100 cells. Data are plotted as mean ± SD. Abbreviations: VAT: visceral adipose tissue, TCR: T cell receptor. See Figure S2 for the correlation between Id3^-^ Tregs and ST2^+^ Tregs across tissues.

To better understand the relationship between ST2 expression and eTreg differentiation, we next analyzed the relative distribution of Tregs expressing ID2 or ID3 and ST2 across tissues. The differentiation of eTregs into a more suppressive and activated phenotype is thought to be a unidirectional terminal differentiation step that is accompanied by the loss of ID3 expression and a corresponding increase in ID2 (Sullivan et al., 2019). Using an Id2 reporter mouse line, we confirmed that tissues in which eTregs predominated, such as VAT, colon, and skin, had higher proportions of Id2^+^ Tregs than tissues with higher frequencies of cTregs, such as blood, spleen, and lungs (Figure 2A). Among the Id2^+^ Tregs, not all co-expressed ST2, and the degree varied by tissue (Figures 2A and 2B). Among ST2^+^ Tregs, about half co-expressed Id2 in blood, spleen, and lungs, and the majority co-expressed Id2 in VAT, colon, and skin (Figures 2A and 2C). Interestingly, the frequency of KLRG1^+^ Tregs was highest in Id2^+^ ST2^+^ Tregs followed by Id2^-^ ST2^+^ Tregs and was lowest in Id2^+^ ST2^-^ and Id2^-^ ST2^-^ Tregs, indicating that KLRG1 expression was more correlated with the expression of ST2 than Id2 (Figure 2D). Using an Id3 reporter line, we confirmed that findings from Id3^-^ Tregs were comparable to those of Id2^+^ Tregs (Figures S2A-S2D).

Collectively, these data suggest that ST2^+^ Tregs constitute a subpopulation of activated Tregs enriched in numerous nonlymphoid tissues as well as in circulation. We found that ST2^+^ Tregs varied substantially in both frequency and phenotype across the tissue types analyzed and that ST2 expression on Tregs in these tissues did not correlate strictly with other markers of Treg differentiation or activation.

### Tissue-specific gene expression patterns dominate over ST2-specific expression patterns

**Figure 3:**
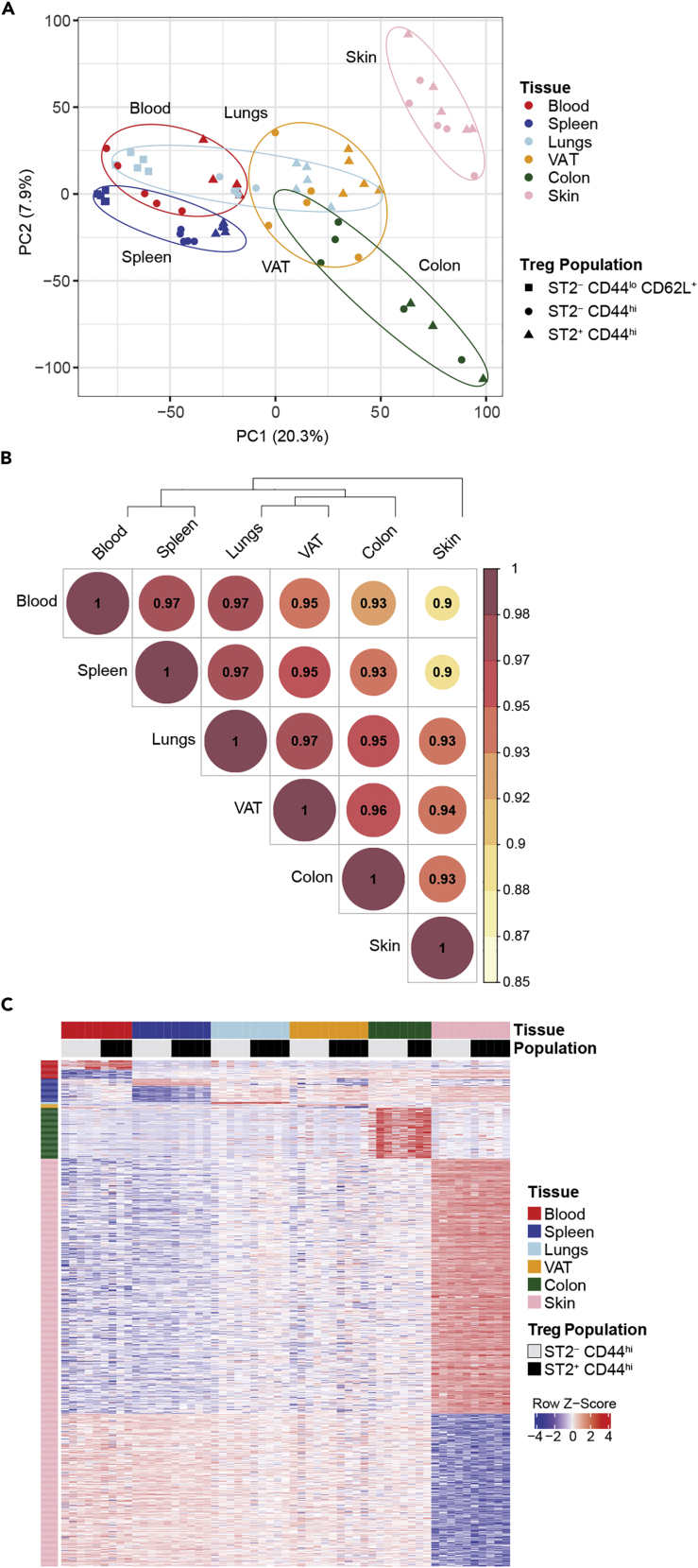
RNAseq analysis of ST2^+^ Tregs and ST2^-^ Tregs across tissues RNAseq was performed on flow sorted ST2^+^ CD44^hi^ Tregs and ST2^-^ CD44^hi^ Tregs from 6 different tissue sites (blood, spleen, lungs, VAT, colon, and back skin) and ST2^-^ CD44^lo^ CD62L^+^ Tregs from spleen and lungs. Data are from 5 male Foxp3^YFP−cre^ mice ages 16–19 weeks old. (A) PCA based on all genes from all populations and tissues. The percentages alongside the PC axes labels are the percentage of variance explained by each PC. (B) Dendrogram and correlation coefficient using Spearman’s ranked-based correlation analysis of all genes in libraries from ST2^+^ CD44^hi^ Tregs and ST2^-^ CD44^hi^ Tregs. (C) Heatmap of pooled tissue-specific DEGs, sorted by tissue. Data displayed as *Z* score. Abbreviations: VAT: visceral adipose tissue, PCA: principal component analysis, DEG: differentially expressed genes. See Figure S3 for the correlation of factors/categorical variables across PC coordinates and Venn diagrams identifying tissue-specific DEGs displayed in Figure 3C.

To better understand the relationship between ST2^+^ and ST2^-^ Tregs across different tissues, we performed RNAseq on sorted populations from blood, spleen, lungs, abdominal VAT, colon, and back skin. ST2^+^ CD44^hi^ Tregs and ST2^-^ CD44^hi^ Tregs were sorted from all six tissues; ST2^-^ CD44^lo^ CD62L^+^ Tregs were also sorted from the spleen and lungs. In principal component analysis (PCA), the tissues were distributed in a gradient along PC1, with blood and spleen at one end, lungs and VAT near the middle, and colon and skin at the other end (Figure 3A). Within most individual tissues, PC1 also separated Treg populations based on ST2 expression. ST2^+^ CD44^hi^ Tregs, ST2^-^ CD44^hi^ Tregs, and ST2^-^ CD44^lo^ CD62L^+^ Tregs each formed distinct clusters within the spleen and lungs; the separation between ST2^+^ Tregs and ST2^-^ Tregs was least apparent in colon and skin (Figure 3A). Tissue type also showed high intraclass correlation across several PC coordinates, indicating that the tissue type was a large contributor to the variance captured by those principal components; combining tissue and Treg population resulted in an even higher correlation (Figure S3A). As expected, variables like mouse ID and measures of library quality such as CV coverage and percent aligned did not show a high correlation across PC coordinates (Figure S3A). When we calculated Spearman’s rho for pairwise comparisons of all tissues, we found that clustering of ranked correlations demonstrated a hierarchy across tissues (Figure 3B). Gene expression in blood Tregs was most similar to those in spleen, followed by lungs, VAT, colon, and then skin.

To further interrogate the differences in transcriptional profiles of Tregs across tissues, we next identified genes that were differentially expressed (DEGs) between Tregs in different tissues independent of ST2 expression status. We performed these analyses on libraries from ST2^+^ and ST2^-^ Tregs separately and then determined the overlap. For example, for blood-specific DEGs from ST2^+^ Tregs, we identified DEGs for blood vs spleen, lungs, VAT, colon, and skin and overlayed these gene sets in a Venn diagram to identify DEGs between ST2^+^ Tregs in blood and ST2^+^ Tregs in all other tissues (unique blood ST2^+^ DEGs). This process was then repeated with ST2^-^ Tregs. Genes unique to ST2^+^ Tregs in the blood and to ST2^-^ Tregs in the blood were then overlayed to identify genes that were unique to Tregs in the blood independent of their ST2 expression. We repeated this analysis for all tissues (Figures S3B–S3D). A heatmap of all tissue unique genes demonstrated that the largest number of tissue-specific DEGs was found in Tregs in the skin, followed by Tregs in the colon (Figure 3C). Overall, the genes that were uniquely upregulated in skin Tregs were expressed at lower levels in blood and spleen, and conversely, genes that were uniquely downregulated in skin Tregs were expressed at higher levels in blood and spleen. DEGs unique to spleen and blood Tregs also showed a reciprocal relationship in expression levels in blood and spleen Tregs compared to skin Tregs.

In summary, our experiments revealed a hierarchical order in the relationship of Treg transcriptomes across tissues and demonstrated a greater role for the tissue microenvironment than ST2 expression in distinguishing the transcriptional profiles of different Treg populations across the tissues examined.

### A small core set of genes define a common ST2^+^ Treg signature across tissues

**Figure 4:**
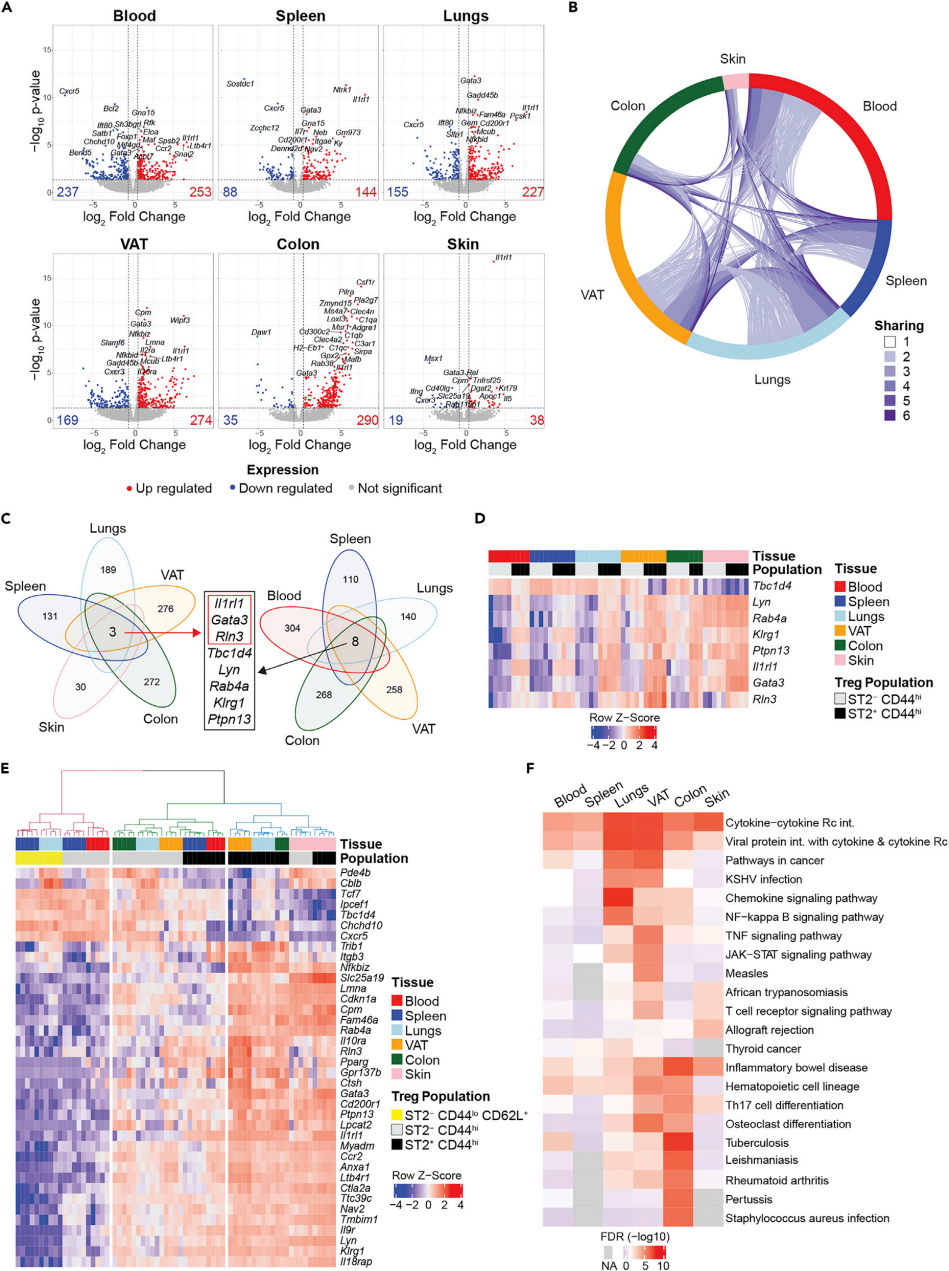
RNAseq analysis of DEGs between ST2^+^ and ST2^-^ Tregs (A) Volcano plots displaying DEGs between ST2^+^ CD44^hi^ Tregs versus ST2^-^ CD44^hi^ Tregs within each tissue. Genes with FC > 1.5 and adjusted p value <0.05 were considered significant. (B) Circos plot using DEGs between ST2^+^ versus ST2^-^ Tregs from individual tissues as assessed in Figure 4A; arcs show DEGs shared between tissues; color code indicates the number of tissues sharing a given DEG. (C) Venn diagrams showing the overlap of DEGs between ST2^+^ versus ST2^-^ Tregs from individual tissues as assessed in Figure 4A across all tissues excluding blood (left) and all tissues excluding skin (right). (D) Heatmap showing gene expression of DEGs between ST2^+^ versus ST2^-^ Tregs that are common to at least 5 of 6 tissues sampled. Three DEGs – *Il1rl1*, *Gata3*, and *Rln3* – are common to all tissues. Data displayed as *Z* score. (E) Heatmap showing hierarchical clustering and gene expression of DEGs between ST2^+^ versus ST2^-^ Tregs that are common to at least 4 of 6 tissues sampled. Data displayed as *Z* score. (F) Heatmap of FDR (-log10) of top KEGG pathways across tissues. The KEGG pathways plotted represent the top 6 terms in each tissue based on FDR. Values in red represent significant pathways (-log10 FDR >1.3 or FDR <0.05); NA = pathways that are not represented in the gene list. Abbreviations: VAT: visceral adipose tissue, DEG: differentially expressed genes, KEGG: Kyoto Encyclopedia of Genes and Genomes Database. See Figure S4 for GATA3 flow cytometric analysis of ST2^+^ and ST2^-^ Tregs across tissues. Figure S5A for expression of core DEGs between ST2^+^ and ST2^-^ Tregs across tissues, Figure S5B for Spearman’s correlation analysis of DEGs between ST2^+^ and ST2^-^ Tregs across tissues, Figure S6 for enriched KEGG pathways in DEGs between ST2^+^ and ST2^-^ Tregs in each tissue, and Table S1 for ST2 Treg RNAseq Datasets.

To compare the transcriptional profiles of ST2^+^ and ST2^-^ Tregs, we identified the DEGs between ST2^+^ and ST2^-^ Tregs in individual tissues (FC > 1.5 and adjusted p value <0.05; Figure 4A). Blood Tregs had the largest number of DEGs between ST2^+^ and ST2^-^ Tregs with nearly 500 genes that were up- or downregulated between these two populations; skin Tregs had the fewest number of DEGs with fewer than 60 genes (Figure 4A; for a full list of DEGs from all tissues see Table S1). When DEGs between ST2^+^ and ST2^-^ Tregs from each tissue were visualized on a circos plot that linked DEGs shared by the various tissues, it was evident that a large number of DEGs were shared across two or three organs but very few were shared by five or more organs. Furthermore, except for the lungs and spleen, more than half of DEGs in a given tissue were unique to that tissue (Figure 4B). Only three DEGs were shared by all the tissues analyzed (Figures 4C and 4D). As expected, expression of *Il1rl1* (ST2), whose surface expression was used to sort ST2^+^ and ST2^-^ Tregs, was significantly higher in ST2^+^ Tregs than ST2^-^ Tregs in all tissues examined. Two additional genes—*Gata3* and *Rln3*—were also more highly expressed in ST2^+^ Tregs than ST2^-^ Tregs in all tissues (Figures 4C and 4D). *Rln3* encodes relaxin 3, an insulin-like hormone that acts as a neuropeptide in the brain but has not been previously described in immune cells (Bathgate et al., 2002). GATA3, the master T_H_2 transcription factor, can regulate ST2 expression and can be upregulated by IL-33/ST2 signaling (Schiering et al., 2014). By flow cytometry, the median fluorescence intensity of GATA3 was significantly higher in ST2^+^ Tregs than ST2^-^ Tregs in blood, spleen, VAT, and colon and trended higher in ST2^+^ Tregs in all tissues analyzed (Figures S4A and S4B). Direct gating on GATA3^+^ cells showed a frequency of at least 70% on ST2^+^ Tregs in all tissues analyzed. A higher frequency of ST2^+^ Tregs than ST2^-^ Tregs expressed GATA3 in all tissues but the skin, where a large majority of both ST2^+^ and ST2^-^ Tregs expressed GATA3 (Figure S4C).

When analyzing DEGs shared by all organs but the skin, the site in which ST2^+^ and ST2^-^ Tregs were most alike, there was a core of 8 genes that were differentially expressed between ST2^+^ and ST2^-^ Tregs (Figures 4C, 4D and S5A). In addition to *Il1rl1*, *Gata3*, and *Rln3*, DEGs in this core signature included *Klrg1*, *Rab4a*, *Lyn*, *Ptpn13*, and *Tbc1d4*. Of these genes, only *Tbc1d4* was downregulated in ST2^+^ Tregs compared to ST2^-^ Tregs. KLRG1 is a co-inhibitory marker on CD8^+^ T cells and NK cells but is also considered to be an activation marker on Tregs (Kornete et al., 2017; Rosenblum et al., 2011; Stephens et al., 2007). Higher expression of KLRG1 on ST2^+^ Tregs was also seen at the protein level (Figure 1D). The remaining four genes in this core signature all encoded gene products that are not well studied in Tregs but are involved in a wide array of intracellular signaling pathways in multiple cell types: LYN, a tyrosine-protein kinase; RAB4A, a small GTPase of the Ras superfamily; PTPN13, a tyrosine phosphatase; and TBC1D4, a Rab GTPase.

We then used a gene list comprised of DEGs between ST2^+^ and ST2^-^ Tregs in at least four tissues to determine whether there were tissue-specific expression patterns within these DEGs. When these 38 genes were visualized in a heatmap (Figure 4E), clustering based on the expression pattern of these genes demonstrated a hierarchical order: ST2^-^ Tregs from the skin were more similar to ST2^+^ Tregs from skin, colon, VAT, and lungs than other ST2^-^ Tregs. In addition, ST2^-^ Tregs from the blood and spleen more closely resembled CD44^lo^CD62L^+^ cTregs than ST2^-^ Tregs from other tissues. Finally, ST2^+^ Tregs from the blood and spleen were more similar to ST2^-^ Tregs from the lungs, VAT, and colon than other tissue ST2^+^ Tregs. These findings suggest a hierarchy in developmental or activation stage in Tregs from spleen and blood progressing toward lungs, VAT, colon, and then skin. Consistent with these findings, ranked correlation coefficients based on DEGs of ST2^+^ Tregs versus ST2^-^ Tregs pooled from all tissues yielded a hierarchical order that was similar to the analysis based on overall gene expression profiles (Figure S5B).

To identify gene sets or pathways that distinguished ST2^+^ Tregs from ST2^-^ Tregs, we performed gene set enrichment analysis (GSEA) of the DEGs in each tissue (Figure 4F) (Ge et al., 2020). “Cytokine-cytokine receptor interaction” was one of the most significantly enriched terms and included a substantial number of chemokine receptors (Figures 4F, S6A and S6B). Some genes in this functional category—including the chemokine receptors *Ccr2* and *Cxcr5* and cytokine receptors or associated signaling components *Il9r*, *Il10ra*, and *Il18rap*—were differentially expressed across many of the tissues examined (Figure S6B).

These data demonstrated that there is a small core set of DEGs between ST2^+^ and ST2^-^ Tregs across all or most tissues analyzed, but that more substantial differences exist between ST2^+^ and ST2^-^ Tregs in individual tissues, particularly the blood. In most cases, more than half of the DEGs were unique to a given tissue. GSEA of DEGs between ST2^+^ and ST2^-^ Tregs highlighted cytokine signaling pathways important in Treg function as well as a large number of chemokine receptors that suggest that ST2^+^ Tregs represent a more migratory subset of Tregs.

### Chemokine receptor analysis identifies ST2^+^ Tregs as a population with high migratory potential

**Figure 5:**
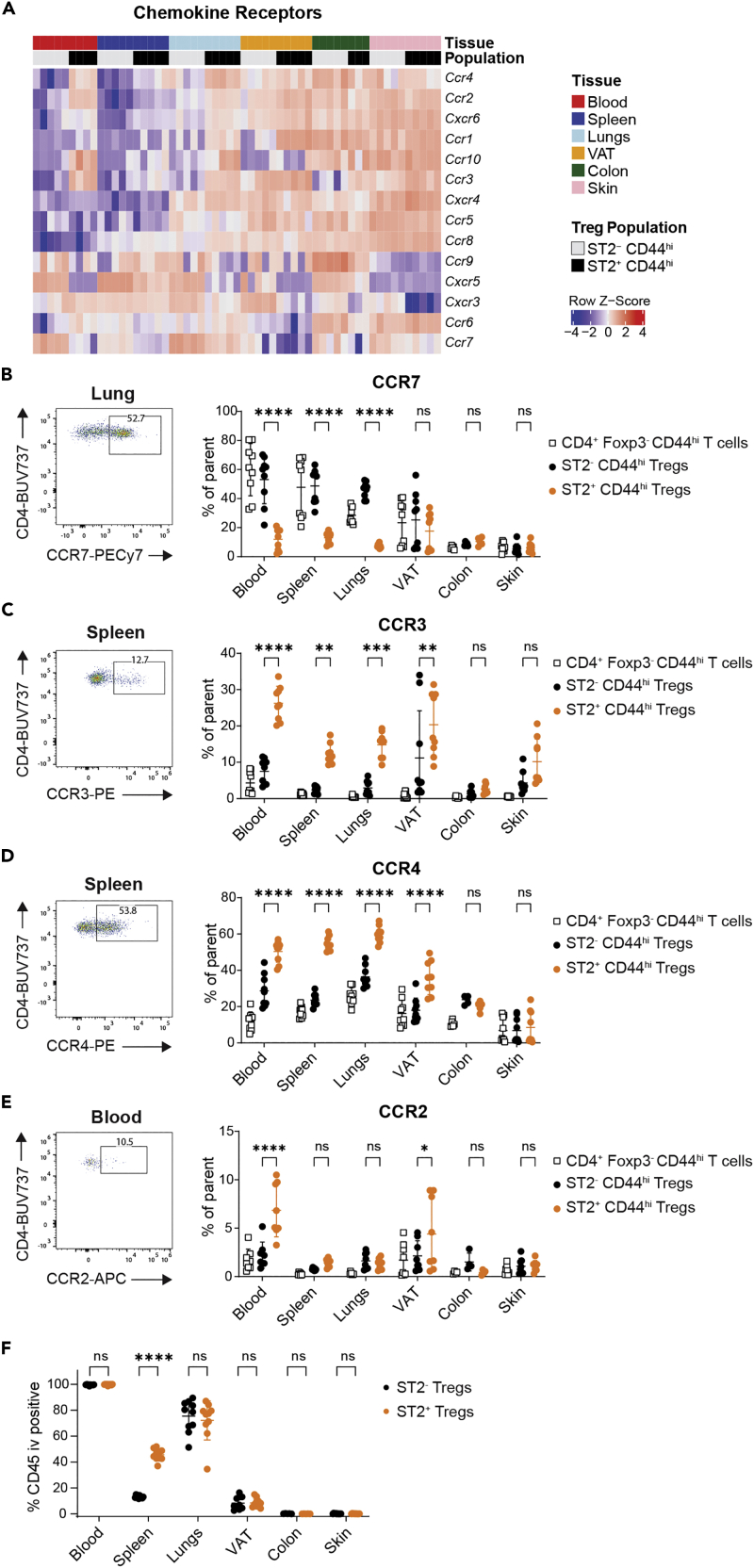
Chemokine receptor expression and intravascular labeling of ST2^+^ and ST2^-^ Tregs (A) Heatmap showing gene expression of chemokine receptors in indicated populations and tissues. Data displayed as *Z* score. (B–E) Representative flow plots and relative frequency of (B) CCR7, (C) CCR3, (D) CCR4, and (E) CCR2 on ST2^+^ CD44^hi^ Tregs, ST2^-^ CD44^hi^ Tregs, and Foxp3^-^ CD4^+^ CD44^hi^ T cells across tissues as assessed by flow cytometry. Lymphocytes were pregated on live singlets, CD45^+^, and TCRβ^+^ CD4^+^ cells. Data are plotted as mean ± SD and pooled from 2 experiments with n = 4–5 for all tissues but colon. For colon, n = 5 in B and D and n = 4 in E, each from one experiment, and n = 7 pooled from 2 experiments in C. Indicated means were compared with two-way ANOVA with Sidak’s multiple comparisons test, ns p > 0.05, ∗p < 0.05, ∗∗p < 0.01, ∗∗∗p < 0.001, ∗∗∗∗p < 0.0001. (F) Frequency of i.v.-labeled CD45 on ST2^+^ and ST2^-^ Tregs in indicated tissues. Mice received 3 μg of anti-CD45 i.v. prior to organ harvesting to evaluate the perivascular localization of cells. Data are plotted as mean ± SD and pooled from 2 experiments of n = 5 each. Indicated means were compared with two-way ANOVA with Sidak’s multiple comparisons test, ns p > 0.05, ∗∗∗∗p < 0.0001. Abbreviations: VAT: visceral adipose tissue, TCR: T cell receptor, CCR: CC chemokine receptor. See Figure S7 for chemokine receptor gene expression profiles of ST2^+^ and ST2^-^ Tregs across tissues.

To further examine how the unique tissue distribution and phenotype of ST2^+^ Tregs might be reflected in the chemokine receptors they express, we first used the RNAseq dataset to look at a variety of chemokine receptors (Figure 5A). Transcriptional profiling of CD44^hi^ Tregs showed that *Ccr7*, which is expressed predominantly by cTregs, was most highly expressed in blood and lung ST2^-^ Tregs and was downregulated in ST2^+^ Tregs compared to ST2^-^ Tregs in most tissues examined (Figures 5A and S7). The expression of *Cxcr5*, a follicular T cell-associated marker, and *Cxcr3*, a T_H_1-associated marker, was lower on ST2^+^ Tregs than on ST2^-^ Tregs across several tissues (Figures 5A and S7). These data further support the association of ST2 expression with eTregs, but also suggest that under homeostatic conditions, ST2 may not be expressed on follicular regulatory T cells (Tfr) or Tregs that specialize in the suppression of T_H_1-type responses.

Several chemokine receptors, for example *Ccr5*, *Ccr8*, *Cxcr4*, and *Cxcr6*, increased in expression across tissues, with low expression in blood Tregs, moderate expression in Tregs from the lungs or VAT, and high expression in skin Tregs (Figures 5A and S7). The expression of *Ccr2*, *Ccr3*, *Ccr4*, and *Ccr10* in circulating ST2^+^ Tregs was higher than in ST2^-^ Tregs in the blood and was comparable to the expression of these chemokine receptors in Tregs in nonlymphoid tissues such as the skin (Figures 5A and S7). We next confirmed the expression patterns of a select panel of these chemokine receptors by flow cytometry. Consistent with the RNAseq data, flow cytometric analysis confirmed that CCR7 was expressed on a low fraction of ST2^+^ Tregs. Even within CD44^hi^ Tregs, a lower frequency of ST2^+^ Tregs than ST2^-^ Tregs expressed CCR7 in spleen, blood, and lungs (Figure 5B). In contrast, both CCR3 and CCR4 were expressed at a higher frequency on ST2^+^ Tregs than on ST2^-^ Tregs in blood, spleen, lungs, and VAT (Figures 5C and 5D). A higher frequency of ST2^+^ Tregs than ST2^-^ Tregs expressed CCR2 in blood and VAT but not in the other tissues analyzed (Figure 5E).

The extraordinary breadth of chemokine receptor expression on ST2^+^ Tregs in the blood suggests that ST2^+^ Tregs may have a high migratory potential. To examine whether ST2^+^ Tregs had a distinct localization within tissues that might also contribute to their migratory potential, we used intravascular (i.v.) CD45 antibody labeling to determine what fraction of Tregs were within or near the vasculature. We found that a larger fraction of ST2^+^ Tregs than ST2^-^ Tregs were i.v. labeled in the spleen (Figure 5F). The majority of Tregs in the lungs were i.v. labeled regardless of ST2 status and very low or no labeling of Tregs was seen in the VAT, colon, and skin. (Figure 5F). Thus, although ST2^+^ and ST2^-^ Treg localization appears similar in most tissues, ST2^+^ Tregs may occupy a distinct, more perivascular niche than ST2^-^ Tregs within the spleen.

### Adoptive transfers reveal differential Treg recovery in tissues and plasticity of ST2 expression

**Figure 6:**
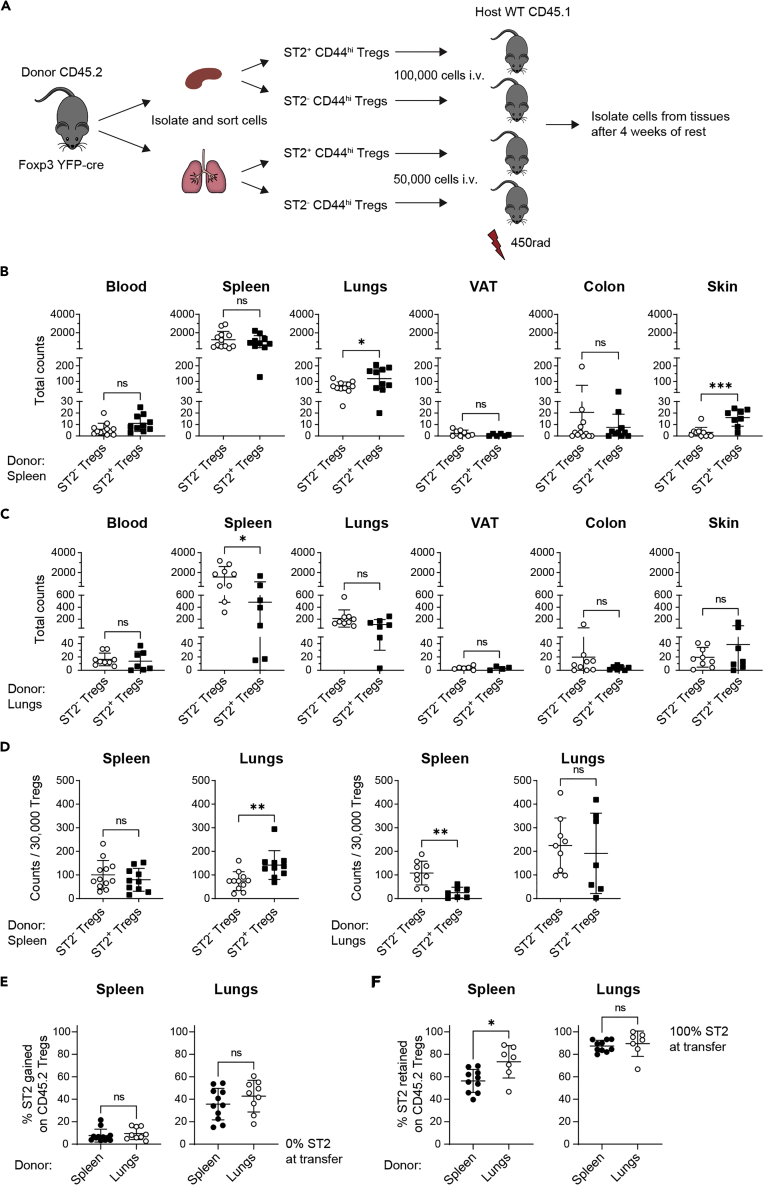
Adoptive transfer of ST2^+^ and ST2^-^ Tregs from spleen or lungs into sublethally irradiated WT hosts (A) Experimental setup: ST2^+^ CD44^hi^ Tregs and ST2^-^ CD44^hi^ Tregs were sorted from either the spleen or lungs of CD45.2 Foxp3^YFP−cre^ mice and transferred i.v. into separate sublethally irradiated CD45.1 hosts. Organs of host mice were harvested 4 weeks after transfer to assess recovery of transferred Tregs. Lymphocytes were gated on live singlets, CD4^+^ FOXP3^+^ CD45.2^+^ cells. (B) Total numbers of CD45.2^+^ Tregs recovered in the indicated tissues after transfer of splenic ST2^-^ CD44^hi^ Tregs or splenic ST2^+^ CD44^hi^ Tregs. (C) Total numbers of CD45.2^+^ Tregs recovered in the indicated tissues after transfer of pulmonary ST2^-^ CD44^hi^ Tregs or pulmonary ST2^+^ CD44^hi^ Tregs. B and C: counts are per whole organ, per 3 × 4 cm back skin or per 450–800 uL blood. (D) CD45.2^+^ Treg counts from B and C were normalized to 30,000 Tregs. (E) Gain of ST2 expression on transferred CD45.2^+^ ST2^-^ Tregs recovered from the indicated tissues. (F) Retention of ST2 expression on transferred CD45.2^+^ ST2^+^ Tregs recovered from the indicated tissues. B-F: Means were compared with unpaired two-tailed test, ns p > 0.05, ∗p < 0.05, ∗∗p < 0.01, ∗∗∗p < 0.001. Data plotted as mean ± SD Data are pooled from 2 to 3 experiments of n = 2–5 mice each. For transferred splenic ST2^-^ Tregs, n = 12 for host blood, spleen, lungs, and colon; n = 10 for skin; n = 8 for VAT. For transferred splenic ST2^+^ Tregs, n = 10 for host blood, spleen, lungs, and colon; n = 8 for skin; n = 6 for VAT. For transferred pulmonary ST2^-^ Tregs, n = 9 for host blood, spleen, lungs, colon, and skin and n = 6 for VAT. And for transferred pulmonary ST2^+^ Tregs, n = 7 for host blood, spleen, lungs, colon, and skin and n = 4 for VAT. Abbreviations: VAT: visceral adipose tissue. See Figure S8 for adoptive transfer of 1∗10^6^ of total ST2^-^ Tregs from the spleen into sublethally irradiated WT hosts.

Since Treg transcriptional signatures were driven predominantly by their tissue of origin rather than whether they expressed ST2, we next used adoptive transfer experiments to assess the capacity of ST2^+^ Tregs and ST2^-^ Tregs from different donor tissues to accumulate in a variety of host tissues. Donor ST2^+^ or ST2^-^ Tregs from either the spleen or the lungs were transferred i.v. into sublethally irradiated, congenically marked hosts. (Figure 6A). First, we observed that ST2^+^ and ST2^-^ Tregs sorted from the spleen (Figure 6B) and the lungs (Figure 6C) were found in a variety of target tissues in addition to their tissue of origin. The total counts of transferred Tregs recovered were highest in spleen, followed by lungs, and then blood, colon, and skin, with very few transferred cells recovered in VAT. (Figures 6B and 6C). Second, both the organ of origin and ST2 expression status dictated Treg recovery in tissues after transfer. If the donor Tregs originated from the spleen, we recovered more Tregs from the host lungs and skin after transfer of ST2^+^ Tregs than ST2^-^ Tregs but equivalent numbers of these two Treg populations from all other host tissues. If the donor Tregs originated from the lungs, we recovered fewer Tregs from the host spleen after transfer of ST2^+^ Tregs than ST2^-^ Tregs but equivalent numbers from all other host tissues (Figures 6B–6D). These differences were even more evident when we normalized the number of recovered donor Tregs to a fixed number of Tregs in the host organ analyzed. Thus, Tregs from the spleen repopulated the spleen independent of their ST2 status, and Tregs from the lungs repopulated the lungs independent of their ST2 status. However, Tregs from the spleen repopulated the lungs preferentially if they were ST2^+^, whereas Tregs from the lungs repopulated the spleen preferentially if they were ST2^-^ (Figure 6D).

Finally, our experiments revealed that transferred ST2^-^ Tregs from both the spleen and lungs acquired ST2 expression at frequencies that paralleled the frequency of ST2 on Tregs within the host organ (Figure 6E). When we transferred a higher number of cells (1∗10^6^ total ST2^-^ Tregs from the spleen) in order to recover higher numbers of transferred Tregs from host tissues, we found that transferred Tregs gained ST2 expression at frequencies equivalent to ST2 on endogenous Tregs in the host spleen, lungs, blood, and skin (Figure S8). After transfer of ST2^+^ Tregs, ST2 expression on donor Tregs found in the host lungs was retained at similar levels regardless of the donor tissue. However, within the host spleen, retention of ST2 expression on donor Tregs from the lungs was higher than on donor Tregs from the spleen. This suggests that at least within the splenic microenvironment, there may be more plasticity of ST2 expression on Tregs originating from the spleen than from the lungs (Figure 6F).

Overall, Tregs from the spleen and the lungs still had the potential to migrate to a variety of host tissues, but both the host tissue and the tissue of origin influenced a Treg’s potential to adapt to a new microenvironment. Our data showed that there is plasticity in Treg ST2 expression and that the tissue-specific frequency of ST2^+^ Tregs appears to be driven predominantly by factors within the target tissue.

### Adoptive transfers into IL-33 KO hosts reveal differential IL-33 dependence of ST2^+^ Tregs derived from lymphoid and nonlymphoid tissues

**Figure 7:**
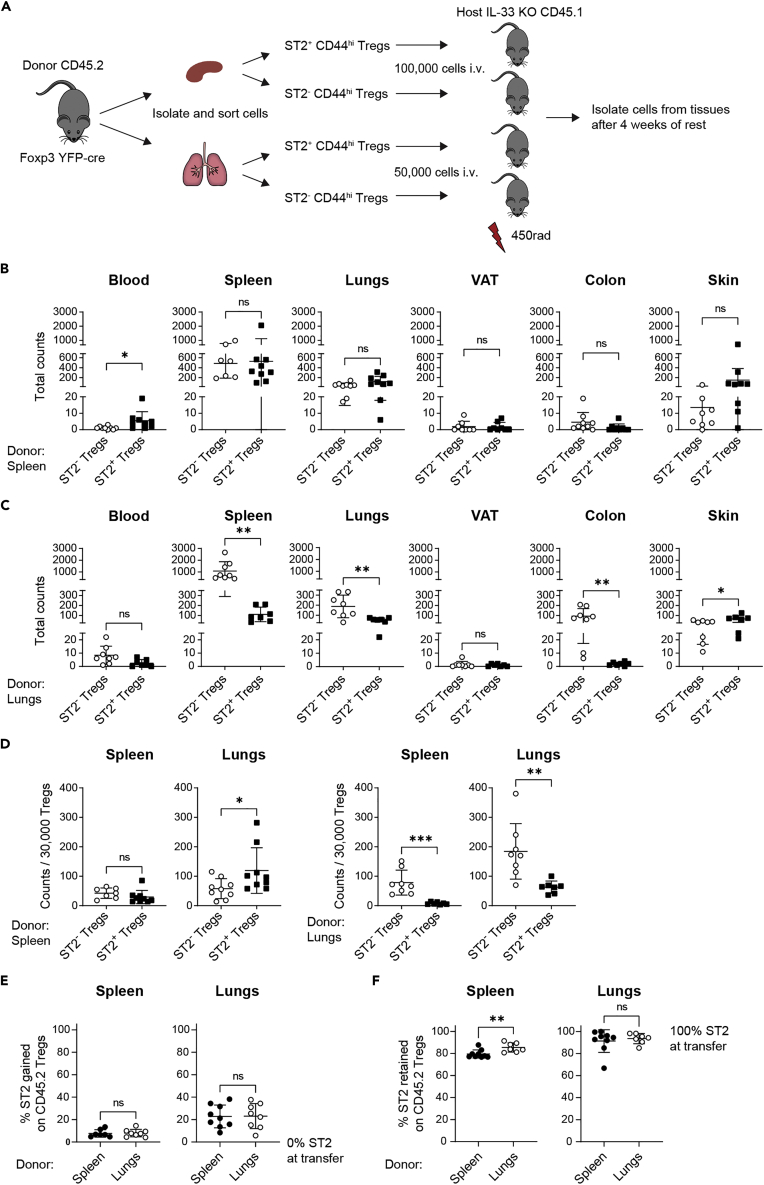
Adoptive transfer of ST2^+^ and ST2^-^ Tregs from spleen or lungs into sublethally irradiated IL-33 KO hosts (A) Experimental setup: ST2^+^ CD44^hi^ Tregs and ST2^-^ CD44^hi^ Tregs were sorted from either the spleen or lungs of CD45.2 Foxp3^YFP−cre^ mice and transferred i.v. into separate sublethally irradiated IL-33 KO CD45.1 hosts. Organs of host mice were harvested 4 weeks after transfer to assess recovery of transferred Tregs. Lymphocytes were gated on live singlets, CD4^+^ FOXP3^+^ CD45.2^+^ cells. (B) Total numbers of CD45.2^+^ Tregs recovered in the indicated IL-33 KO tissues after transfer of splenic ST2^-^ CD44^hi^ Tregs or splenic ST2^+^ CD44^hi^ Tregs. (C) Total numbers of CD45.2^+^ Tregs recovered in the indicated IL-33 KO tissues after transfer of pulmonary ST2^-^ CD44^hi^ Tregs or pulmonary ST2^+^ CD44^hi^ Tregs. B and C: counts are per whole organ, per 3 × 4 cm back skin or per 300–800 uL blood. (D) CD45.2^+^ Treg counts from B and C were normalized to 30,000 Tregs. (E) Gain of ST2 expression on transferred CD45.2^+^ ST2^-^ Tregs recovered from the indicated tissues. (F) Retention of ST2 expression on transferred CD45.2^+^ ST2^+^ Tregs recovered from the indicated tissues. B-F: Means were compared with unpaired two-tailed test, ns p > 0.05, ∗p < 0.05, ∗∗p < 0.01, ∗∗∗p < 0.001. Data plotted as mean ± SD Data are pooled from 3 to 4 experiments. For transferred splenic ST2^-^ Tregs, n = 7 for host spleen; n = 8 for host VAT and skin; n = 9 for host blood, lung, and colon. For transferred splenic ST2^+^ Tregs, n = 9 for host blood, spleen, lungs, colon, and skin; n = 8 for VAT. For transferred pulmonary ST2^-^ Tregs, n = 8 for all host tissues. And for transferred pulmonary ST2^+^ Tregs, n = 7 for all host tissues. Abbreviations: VAT: visceral adipose tissue.

We next determined whether lack of IL-33 in the host would affect the ability of ST2^+^ Tregs to accumulate in host tissues. Donor ST2^+^ or ST2^-^ Tregs from either the spleen or the lungs were transferred i.v. into sublethally irradiated, congenically marked IL-33 KO hosts (Figure 7A). As in wild-type hosts, ST2^+^ and ST2^-^ Tregs from the spleen (Figure 7B) and the lungs (Figure 7C) were found in a variety of IL-33 KO target tissues in addition to their tissue of origin. ST2^+^ and ST2^-^ Tregs originating from donor spleen accumulated in IL-33 KO host tissues in a pattern that paralleled that of wild-type hosts (Figures 7B and 7D). However, the accumulation of ST2^+^ and ST2^-^ Tregs from donor lungs was different between wild-type and IL-33 KO hosts. In wild-type hosts, donor ST2^+^ and ST2^-^ Tregs from the lungs accumulated in comparable numbers in all tissues but the spleen, where fewer ST2^+^ than ST2^-^ Tregs where found (Figures 6C and 6D). In IL-33 KO hosts, this difference was still observed in the host spleen (Figures 7C and 7D). However, in contrast to what was seen in wild-type hosts, there were also fewer ST2^+^ than ST2^-^ Tregs from the lungs that accumulated in IL-33 KO host lungs and colon (Figures 7C and 7D). In the IL-33 KO host skin, higher numbers of ST2^+^ than ST2^-^ Tregs were recovered after transfer, despite the lack of IL-33 (Figure 7C).

When we examined the gain of ST2 expression on donor ST2^-^ Tregs and the retention of ST2 expression on donor ST2^+^ Tregs, the results in IL-33 KO hosts were comparable to those of wild-type hosts (Figures 7E and 7F). Transferred ST2^-^ Tregs from both the spleen and lungs acquired ST2 expression at frequencies comparable to the frequency of ST2 on Tregs within the host organs (Figure 7E). ST2 expression on donor ST2^+^ Tregs found in the host lungs was retained at similar levels regardless of the donor tissue. And as was seen in the wild-type host spleen, retention of ST2 expression on donor Tregs from the lungs was higher than on donor Tregs from the spleen.

These data demonstrate that the accumulation of donor ST2^+^ and ST2^-^ Tregs in host tissues is not dependent on IL-33 when the donor organ is the spleen. Fewer donor ST2^+^ Tregs than ST2^-^ Tregs from the lungs accumulated in the spleen, and this was also independent of host IL-33 expression. However, donor ST2^+^ Tregs from the lungs had a decreased capacity to accumulate in IL-33 KO lungs and colon compared to donor ST2^-^ Tregs from the lungs, suggesting that IL-33 signaling affects the accumulation of ST2^+^ Tregs in nonlymphoid organs such as the lungs and colon. Furthermore, plasticity of ST2 expression is dictated by the target tissue and tissue of origin but is not dependent on IL-33.

## Discussion

ST2 has emerged as a marker for tissue Tregs, but the relationship between ST2^+^ and ST2^-^ Tregs across and within different tissues remains poorly defined. Whether the expression of ST2 on Tregs represents a distinct developmental step or is driven by tissue-specific signals is also still unknown. In the present study, we identify unique characteristics of ST2^+^ Tregs and demonstrate a hierarchy in tissue-specific gene and protein expression in Tregs that may define distinct activation and differentiation states of Tregs across different tissues. Few genes were differentially expressed between ST2^+^ and ST2^-^ Tregs universally across all tissues, but there were large differences in the transcriptional profiles of ST2^+^ and ST2^-^ Tregs within individual tissues. Thus, ST2-specific Treg signatures appear to be driven by local tissue microenvironments, reflective of specific functions for ST2^+^ Tregs in individual tissues.

Consistent with previous reports in the literature linking ST2 expression to activated tissue Tregs, we found that ST2^+^ Tregs were all CD44^hi^ across all the tissues we analyzed. In addition, ST2^+^ Tregs were enriched in more activated, differentiated Treg populations such as ID2^+^ and ID3^-^ Tregs. Yet, ST2^+^ Tregs were also present in “earlier” stages of differentiation/activation and included populations of ST2^+^ CD44^hi^ CD62L^+^ Tregs and ST2^+^ ID2^-^ Tregs. Graded levels of activation or development were also reflected within ST2^+^ Treg populations across tissues, since ST2^+^ Tregs in the blood, spleen, and lungs expressed ID2 at lower frequencies than ST2^+^ Tregs in the VAT, colon, and skin. It was notable that only select activation markers such as KLRG1 were consistently correlated with ST2 expression across the tissues examined. Thus, ST2 expression was not strictly correlated with the terminal differentiation of eTregs and may be driven by factors other than those that drive Treg differentiation and activation. Cross-regulation of gene expression of ST2 and GATA3 can occur (Schiering et al., 2014), and GATA3 had a similar expression profile to ST2 across all the tissues we analyzed. ST2 expression may therefore be a part of a GATA3-driven T_H_2-type transcriptional program. Indeed, epigenetic modifications in tissue Tregs in the skin and VAT showed a bias toward genes overexpressed in T_H_2 cells when compared to Tregs from the LN (Delacher et al., 2017). However, we found that GATA3 expression was also high in skin ST2^-^ Tregs, suggesting that, at least in some contexts, high GATA3 expression in Tregs is not sufficient to drive high ST2 expression.

Whereas the expression of ID2/ID3 and CD49b are thought to mark unidirectional developmental stages of Tregs (Fan et al., 2018; Sullivan et al., 2019), we have demonstrated that plasticity exists in ST2 expression on Tregs in tissues. Our transfer experiments suggest that in lymphopenic conditions, local signals from the seeded host tissues as well as tissue-specific imprinting of donor Tregs regulated plasticity in ST2 expression. Although a majority of transferred ST2^+^ Tregs maintained ST2 expression after transfer, a higher fraction of transferred ST2^+^ Tregs originating from the spleen than the lungs lost ST2 expression in the host spleen. The increased stability of ST2 expression on donor ST2^+^ Tregs from the lungs after transfer may reflect a more activated or differentiated state and thus less plasticity of Tregs in nonlymphoid tissues. Notably, whereas the pattern of accumulation of ST2^+^ and ST2^-^ Tregs originating from donor spleen was comparable between wild-type and IL-33 KO hosts, there were distinct differences between the recovery of Tregs in tissues from wild-type and IL-33 KO hosts when the Tregs originated from the lungs. Accumulation of Tregs from donor lungs was not dependent on IL-33 in the host skin, a tissue which may provide a particularly unique microenvironment for Tregs and harbors ST2^+^ and ST2^-^ Tregs that are very transcriptionally similar to one another. However, ST2^+^ Tregs from donor lungs had a decreased capacity to repopulate the lungs and colon in IL-33 KO hosts compared to their ST2^-^ Treg counterparts. IL-33 has been shown to regulate local tissue accumulation of ST2^+^ Tregs in specific contexts (Hung et al., 2020; Kuswanto et al., 2016; Vasanthakumar et al., 2015). Our transfer data suggest that once ST2^+^ Tregs reside in nonlymphoid tissues such as the lungs, additional imprinting may occur that confers on these Tregs an increased dependence on IL-33.

Our data also suggest that a specific tissue niche or milieu can dictate different frequencies of ST2^+^ Tregs in distinct tissues since transferred ST2^-^ Tregs gained ST2 expression at frequencies that matched host organs regardless of the origin of the donor cells. In line with this finding, the transcriptomes of ST2^+^ Tregs in many tissues were more closely related to their ST2^-^ counterparts in the same tissue than to ST2^+^ Tregs in other tissues. In addition, the high frequency of ST2^-^ Tregs in most nonlymphoid tissues and the lack of a large universal ST2^+^ Treg signature across tissues could suggest that Tregs gain ST2 expression once they enter a specific tissue. Using TCR transgenic mice, Li et al. showed that acquisition of the tissue phenotype of Tregs in VAT involved a small intermediate PPARγ^lo^ population in the spleen but required residence within the VAT to gain the full VAT-Treg phenotype (Li et al., 2018). At least at the mRNA level, ST2 was already upregulated in PPARγ^lo^ vs PPARγ^−^ Tregs in the spleen, and PPARγ^lo^ cells more efficiently gave rise to VAT Tregs in transfer experiments. Given that Tregs can gain or lose ST2 expression, it is likely that upregulation of ST2 on Tregs may occur both peripherally and within the tissue of residence.

Both the lungs and the spleen are highly vascularized organs, and the lungs have a particularly unique architecture with a large capillary network in very close proximity to alveoli. Through intravascular antibody labeling, we demonstrated that a higher frequency of ST2^+^ than ST2^-^ Tregs is near vasculature in the spleen, and that a majority of Tregs, regardless of ST2 expression, are perivascular in localization in the lungs. These data suggest that ST2^+^ Tregs may be enriched in the vascular beds in these tissues and may be a more migratory population. We cannot rule out the presence of circulating Tregs in the pulmonary and splenic Tregs we evaluated; however, Tregs from the lungs and spleen were unlikely to solely represent circulating Tregs, given the distinct transcriptional profiles of cells from each site and the differences in behavior of Tregs from the lungs and spleen in our adoptive transfer studies. The i.v. labeling data could indicate that there is exchange of ST2^+^ Tregs between the blood and the spleen and lungs. Indeed, when we examined the hierarchical clustering of ST2^+^ Treg DEGs and the expression of functional markers, we found a bifurcation in expression and phenotype between ST2^+^ Tregs from the blood, spleen, and lungs and those from the VAT, colon, and skin. The decreased ability of ST2^+^ Tregs from the lungs to accumulate in the lungs of IL-33 KO hosts when compared to their ST2^-^ counterparts suggests that lung-specific imprinting occurred, regardless of whether these ST2^+^ Tregs reside within the pulmonary vasculature, parenchyma, or adventitial space.

It is notable that although ST2 has been thought of as a marker for tissue Tregs, nearly a third of circulating Tregs expressed ST2. ST2^+^ Tregs in the blood had significantly higher levels of expression of Ccr2, Ccr3, Ccr4, and Ccr10 than ST2^-^ Tregs in the blood, and expression of these chemokine receptors on circulating ST2^+^ Tregs was comparable to their expression in nonlymphoid tissue Tregs. Thus, circulating ST2^+^ Tregs may constitute a population that recently exited tissue or are poised to enter tissue. Using adoptive Treg transfer studies to introduce traceable Tregs into the bloodstream, we showed that both ST2^+^ Tregs and ST2^-^ Tregs have the potential to seed multiple organs after transfer. As measured by Ki-67 expression, ST2^+^ Tregs from the blood also had the highest levels of proliferation among ST2^+^ and ST2^-^ Tregs from all the tissues examined. Although eTregs are more proliferative than cTregs (Smigiel et al., 2014), we found that less than 20% of Tregs in the colon and skin expressed Ki-67. In addition, studies have shown that there is loss of non-host chimerism of Tregs in the colonic lamina propria after separation of parabionts (Luo et al., 2016), indicating that these Tregs are not locally maintained over long periods of time. Our data suggest that replenishment of tissue Tregs by highly proliferative, circulating ST2^+^ Tregs can be an important mechanism for maintaining Tregs in nonlymphoid tissues. The accumulation and maintenance of eTregs in lymphoid and nonlymphoid sites may thus require a balance of migration from the periphery, survival, and proliferation that is tissue specific.

Taken together, our comprehensive profiling and transfer experiments suggest a model of both ST2-specific and tissue-specific imprinting under homeostatic conditions. In the future, additional studies, such as those that use fate-mapping techniques can be used to address imprinting of tissue Tregs and plasticity in ST2^+^ Treg phenotypes and function under inflammatory conditions and in different disease states.

## Limitations of the study

While our studies have demonstrated substantial heterogeneity in Tregs across different tissues, the bulk RNA sequencing used in these studies only allows for limited conclusions that cannot account for the full diversity of Treg populations that is known to exist at the single cell level and does not support trajectory analysis to investigate developmental relationships. Given that some circulating Tregs may remain in the blood vessels after perfusion, further work will also be necessary to differentiate between distinct ST2^+^ Treg populations that provide specific functions within or nearby the pulmonary vasculature and those that are in circulation. Another limitation of these studies was the low total number of ST2^+^ Tregs that could be isolated from blood and some nonlymphoid tissues; this limitation prevented us from using these very interesting populations as donor cells in the Treg transfer studies. In addition, differences exist in the expression of ST2 on murine Tregs and human Tregs (Lam et al., 2019); thus, the extent to which our studies in the murine system can be translated to humans should be carefully evaluated.

## STAR★Methods

### Key resources table

**Table:** 

REAGENT or RESOURCE	SOURCE	IDENTIFIER
**Antibodies**		

BUV395 Rat Anti-Mouse CD45 Clone 30-F11	BD Biosciences	Cat# 564279; RRID:AB_2651134
BUV737 Rat Anti-Mouse CD4 Clone RM4-5	BD Biosciences	Cat# 612843; RRID:AB_2870165
BUV737 Rat Anti-Mouse CD4 Clone GK1.1	BD Biosciences	Cat# 612761; RRID:AB_2870092
BV421 Rat Anti-Mouse IL-33R (ST2) Clone U29-93	BD Biosciences	Cat# 566309; RRID:AB_2744489
BV605 anti-mouse CD4 Clone 4–5	BioLegend	Cat# 100547; RRID:AB_11125962
BV711 anti-mouse CD8a Antibody	BioLegend	Cat# 100748; RRID:AB_2562100
BV605 anti-mouse TCRβ Clone H57-596	BioLegend	Cat# 109241; RRID:AB_2629563
BV785 anti-mouse CD25 Clone PC61	BioLegend	Cat# 102051; RRID:AB_2564131
APC anti-mouse CD19 Antibody Clone 1D3/CD19	BioLegend	Cat# 152410; RRID:AB_2629839
PE anti-mouse CD45.2 Antibody Clone 104	BioLegend	Cat# 109808; RRID:AB_313445
PE/Cy7 anti-mouse CD45.2 Antibody Clone 104	BioLegend	Cat# 109830; RRID:AB_1186098
BV650 anti-mouse CD44 Antibody Clone IM7	BioLegend	Cat# 103049; RRID:AB_2562600
PE/Cy7 anti-mouse/human KLRG1 (MAFA) Antibody Clone 2F1/KLRG1	BioLegend	Cat# 138416; RRID:AB_2561736
PE anti-mouse CD193 (CCR3) Clone J073E5	BioLegend	Cat# 144506; RRID:AB_2561534
PE anti-mouse CD194 (CCR4) Clone 2G12	BioLegend	Cat# 131204; RRID:AB_1236367
PerCP/Cy5.5 anti-mouse CD103 Antibody Clone 2E7	BioLegend	Cat# 121416; RRID:AB_2128621
AF700 anti-mouse CD62L Antibody Clone MEL-14	BioLegend	Cat# 104426; RRID:AB_493719
Alexa Fluor 488 FOXP3 Monoclonal Antibody Clone FJK-16s	eBioscience	Cat# 53-5773-82; RRID:AB_763537
APC CD45.1 Monoclonal Antibody Clone A20	eBioscience	Cat# 17-0453-82; RRID:AB_469398
Alexa Fluor 700 CD45.2 Monoclonal Antibody Clone 104	eBioscience	Cat# 56-0454-82; RRID:AB_657752
APC CD11b Monoclonal Antibody Clone M1/70	eBioscience	Cat# 17-0112-82; RRID:AB_469343
PE/Cy7 Ki-67 Monoclonal Antibody Clone SolA15	eBioscience	Cat# 25-5698-82; RRID:AB_11220070
PerCP-eFluor710 CD90.2 Monoclonal Antibody Clone 30-H12	eBioscience	Cat# 46-0903-82; RRID:AB_10670882
PE CD103 Monoclonal Antibody Clone 2E7	eBioscience	Cat# 12-1031-82; RRID:AB_465799
APC-eFlour780 CD62L Monoclonal Antibody Clone MEL-14	eBioscience	Cat# 47-0621-82; RRID:AB_1603256
Biotin CD197 (CCR7) Monoclonal Antibody Clone 4B12	eBioscience	Cat# 13-1971-82; RRID:AB_466642
PE Gata-3 Monoclonal Antibody Clone TWAJ	eBioscience	Cat# 12-9966-42; RRID:AB_1963600
Mouse CCR2 APC conjugated Antibody Clone 475301	R&D Systems	Cat# FAB5538A; RRID:AB_10645617

**Chemicals, peptides, and recombinant proteins**		

Collagenase IV	Sigma Aldrich	Cat# C5138-1G
DNase I	Sigma Aldrich	Cat# DN25-1G
PE/Cy7 Streptavidin	BioLegend	Cat# 405206

**Critical commercial assays**		

Foxp3/Transcription Factor Staining Buffer Set	eBioscience	Cat# 00-5523-00
Zombie Aqua Fixable Viability Kit	Biolegend	Cat# 423102
Zombie NIR Fixable Viability Kit	Biolegend	Cat# 423106
CD4^+^ T Cell Isolation Kit	Miltenyi Biotec	Cat# 130-104-454
SMART-Seq v4 Ultra Low Input RNA Kit for Sequencing	Takara	Cat# 634891
NexteraXT DNA sample preparation kit	Illumina	Cat# FC-131-1002

**Deposited data**		

RNAseq data	This paper	GEO: GSE182322

**Experimental models: Organisms/strains**		

Mouse: B6.129(Cg)-Foxp3^tm4(YFP/icre)Ayr^/J (Foxp3^YFP-Cre^)	The Jackson Laboratory	Stock No: 016959; RRID:IMSR_JAX:016959
Mouse: B6.SJL-Ptprc^a^ Pepc^b^/BoyJ (CD45.1)	The Jackson Laboratory	Stock No: 002014; RRID:IMSR_JAX:002014
Mouse: C57BL/6-Foxp3^tm1Flv^/J (Foxp3^mRFP^)	The Jackson Laboratory	Stock No: 008374; RRID:IMSR_JAX:008374
Mouse: Foxp3^mRFP^Id2^YFP^ mice	Lab of Daniel Campbell	N/A
Mouse: Foxp3^mRFP^Id3^GFP^ mice	Lab of Daniel Campbell	N/A
Mouse: Il33^tm1Amgn^ (IL-33 KO)	Amgen	RRID:MGI:5476738

**Software and algorithms**		

FlowJo	BD Biosciences	flowjo.com
SpectroFlo	Cytek BIosciences	cytekbio.com/pages/spectro-flo
BD FACSDiva	BD Biosciences	bdbiosciences.com
Prism	GraphPad Software	www.graphpad.com
R package edgeR (v3.26.8)	Bioconductor	bioconductor.org
ShinyGO v0.74	South Dakota State University, Xijin Ge Laboratory	bioinformatics.sdstate.edu/go/

### Resource availability

#### Lead contact

Further information and requests for resources and reagents should be directed to and will be fulfilled by the lead contact, Steve Ziegler (sziegler@benaroyaresearch.org).

#### Materials availability

This study did not generate new unique reagents.

### Experimental models and subject details

#### Mice

Foxp3^YFP−cre^ mice (B6.129(Cg)-Foxp3^tm4(YFP/icre)Ayr^/J; Stock No: 026959) and CD45.1 mice (B6.SJL-Ptprc^a^ Pepc^b^/BoyJ; Stock No: 002014) were purchased from The Jackson Laboratory (Janowska-Wieczorek et al., 2001; Rubtsov et al., 2008). Foxp3^mRFP^ mice (C57BL/6-Foxp3^tm1Flv^/J; JAX Stock No: 008374), Foxp3^mRFP^Id2^YFP^ mice, and Foxp3^mRFP^Id3^GFP^ mice were obtained from Daniel Campbell (Miyazaki et al., 2011; Yang et al., 2011). Interleukin-33 deficient mice (IL-33 KO) were provided by Dirk Smith (Amgen) and bred to CD45.1 mice (Martin et al., 2013). All mice were bred and maintained under SPF conditions in an American Association for the Accreditation of Laboratory Animal Care (AAALAC)-accredited animal facility at the Benaroya Research Institute (BRI). All experiments were performed in accordance with protocols approved by the BRI Animal Care and Use Committee. Age -and sex-matched male and female mice (littermates where applicable) between seven and twenty-six weeks of age were used in all experiments.

### Method details

#### Tissue processing and cell isolation

Mice were euthanized using tribromoethanol (TBE) followed by exsanguination or thoracotomy. Blood was drawn by cardiac puncture and collected in Microvette 200 LH tubes (Sarstedt). In all experiments when lungs were processed, the pulmonary and systemic circulation was then perfused with PBS. Red blood cells in the blood samples were lysed for 5 minutes on ice in RBC lysis buffer (155 mM NH_4_Cl, 11 mM KHCO_3_, 1.3 mM EDTA), washed with PBS, and filtered through a 35 μM strainer. Spleens and LNs were homogenized by mechanical disruption, filtered through a 70 μm cell strainer, and washed with PBS. Splenocytes were then incubated with RBC lysis buffer for 5 minutes on ice to lyse RBCs, filtered through a 70 μm cell strainer, and washed with PBS. The remaining tissues were processed as follows:

##### Colon

The colon was separated from mesenteric fat, and the luminal mucus and fecal matter were removed mechanically at the time of dissection. The colon was transected longitudinally and bisected then washed three times by vortexing followed by transfer into fresh conditioned media (CM; Ca^2+^-free Mg^2+^-free HBSS (Hyclone)/5% FCS (Sigma)). The tissue pieces were incubated with gentle mixing in CM with 1 mM DTT and 5 mM EDTA for 15 min at 37°C and then in CM with 5 mM EDTA for 30 min at 37°C. The tissue was then cut into small pieces and digested in 0.4 mg/mL collagenase IV (from *Clostridium histolyticum*; Sigma Aldrich), 0.1 mg/mL DNAse (Sigma), and 10% FCS in HBSS (with Ca^2+^ and Mg^2+^, Hyclone) for 30 min at 37°C. After digestion, the samples were homogenized using a syringe and 18 gauge needle and filtered through a 70 μm cell strainer.

##### Lungs and adipose tissue

Tissues were collected in cold PBS and then cut into small pieces and digested in 0.4 mg/mL collagenase IV (Sigma Aldrich), 0.1 mg/mL DNAse and 10% FCS in HBSS (with Ca^2+^ and Mg^2+^, Hyclone) for 30 min at 37°C. The samples were then homogenized using a syringe and 18 gauge needle and filtered through a 70 μm cell strainer. Cells were then incubated with RBC lysis buffer for 5 minutes on ice, filtered through a 70 μm cell strainer, and washed with PBS.

##### Skin

Shaved back skin (approx. 4 × 3 cm) was cut into small pieces and digested in 1 mg/mL collagenase IV (Sigma Aldrich), 0.1 mg/mL DNAse, 10% FCS, and 25 mM HEPES in RPMI (Sigma) for 90 minutes at 37°C with gentle mixing. The samples were then homogenized using a syringe and 18 gauge needle and filtered twice through a 70 μm cell strainer.

#### Flow cytometry and cell sorting

Flow cytometry was performed using standard methods (Cossarizza et al., 2019). For all fluorochrome-conjugated antibodies optimal concentrations were determined using titration experiments. The following antibodies were obtained from BD, BioLegend, R&D, or eBioscience: CD4 (GK1.5), CD8a (53-6.7), CD11b (M1/70), CD19 (1D3), CD25 (PC61), CD44 (IM7), CD45 (30-F11), CD45.1 (A20), CD45.2 (104), CD62L (MEL-14), CD90.2 (30-H12), CD103 (2E7), FOXP3 (FJK-16s), GATA3 (TWAJ), KLRG1 (2F1/KLRG1), Ki-67 (SolA15), ST2 (U29-93), TCRβ (H57-597), CCR2 (475301), CCR3 (J073E5), CCR4 (2G12), CCR7 (4B12).

Cells were stained with Aqua or Near-IR fixable viability dye according to the manufacturer’s instructions (BioLegend), and then surface stained with an antibody cocktail in FACS buffer (PBS/2% FCS) for 20 minutes at 4°C. For intracellular and intranuclear staining, cells were fixed and permeabilized using the eBioscience FOXP3 fix/perm buffer according to the manufacturer’s instructions and then stained in FOXP3 perm buffer (eBioscience) for 20–60 minutes at 4°C. For studies using Foxp3^YFP−cre^ mice, if other intracellular or intranuclear markers were used in the panel, loss of YFP was confirmed and FOXP3 was stained with FOXP3 AF488 (FJK-16s). For intranuclear GATA3 staining, cells were fixed and permeabilized using the eBioscience FOXP3 fix/perm buffer for 30mins and then stained in FOXP3 perm buffer (eBioscience) for 60 minutes at 4°C. For CCR staining cells were rested at 37°C for 1–2 hours in RPMI +10% FCS prior to CCR surface staining at 37°C.

In general, cells were gated based on FSC-Area and SSC-Area to exclude debris; doublets were excluded by FSC-Area vs. FSC-Height gating; and dead cells were excluded using a fixable viability dye. Flow cytometry was performed on a five laser BD LSR II with FACS Diva Software or a five laser Cytek Aurora with SpectroFlow Software. Data analysis was performed using FlowJo 10.0.x (BD Biosciences).

Cell sorting was performed on a BD FACSAria II or BD FACSAria Fusion Sorter using a 70 μm nozzle.

For RNAseq analysis, blood, spleen, lungs, VAT, colon, and back skin of CD45.2 Foxp3^YFP−cre^ mice were harvested and single cell suspensions were obtained as described above. For spleen and lungs, CD4^+^ T cells were enriched using a CD4^+^ T Cell Isolation Kit according to the manufacturer’s instructions (Miltenyi Biotec) and then surface stained in MACS buffer for 20 minutes at 4°C. Cells were stained with Near-IR fixable viability dye prior to sorting. Live, single, CD45.2^+^, CD8^−^, CD19^−^, CD11b^−^, CD4^+^, YFP^+^, ST2^+^ CD44^hi^ cells and live, single, CD45.2^+^, CD8^−^, CD19^−^, CD11b^−^, CD4^+^, YFP^+^, ST2^-^ CD44^hi^ cells were sorted from all tissues; live, single, CD45.2^+^, CD8^−^, CD19^−^, CD11b^−^, CD4^+^, YFP^+^, ST2^-^ CD44^lo^ CD62L^+^ cells were sorted from spleen and lungs. Cells were collected in 1.5 mL tubes containing lysis buffer (see below). Sort purities for lungs were checked at the beginning of the sort and for spleen at the end of the sort and were routinely higher than 95%.

For Treg transfer experiments, lungs and spleen from 6-8 CD45.2 Foxp3^YFP−cre^ mice were harvested and single cell suspensions were obtained as described above. For splenocytes, CD4 T^+^ cells were enriched using a CD4^+^ T Cell Isolation Kit (Miltenyi Biotec) and then surface stained in FACS buffer for 20 minutes at 4°C. Cell were then stained with Near-IR fixable viability dye prior to sorting. Live, single, CD45.2^+^, CD8^−^, CD19^−^, CD11b^−^, CD4^+^, YFP^+^ cells that were ST2^+^ CD44^hi^, ST2^-^ CD44^hi^, or ST2^-^ (total ST2^-^ Tregs) were sorted and collected in 15 mL conical tubes containing RPMI +10% FCS. Sort purities were checked for all populations sorted and were routinely higher than 95%.

**Table:** 

Optical configuration of LSR II		
Laser	Filter	Used dyes
UV 355nm	740/35	BUV 737
	450/50	DAPI
	379/28	BUV395
Violet 405nm	780/60	Brilliant Violet 785
	710/50	Brilliant Violet 711
	655/8	Brilliant Violet 650
	605/12	Brilliant Violet 605
	525/50	AmCyan, Zombie Aqua, BV510
	450/50	PacificBlue, BV421
Blue 488nm	710/50	PerCP/PerCP-Cy5.5
	525/50	FITC, GFP, YFP
	488/10	SSC
Yellow/Green 561nm	780/60	PE-Cy7
	660/20	PE-Cy5
	620/10	PE-Texas Red, PE-CF594
	582/15	PE, tdTomato
Red 640nm	780/60	APC-Cy7, Zombie NIR
	730/45	AlexaFluor700
	670/30	APC

#### Adoptive transfer experiments

For Treg transfers, spleens and lungs were harvested from CD45.2 Foxp3^YFP−cre^ mice. Cells were resuspended in MACS buffer (PBS/0.5% FCS/2 mM EDTA), and for splenocytes, CD4^+^ T cells were enriched using a CD4^+^ T cell isolation kit (Miltenyi Biotec). After surface staining with antibodies, ST2^+^ CD44^hi^ Tregs and ST2^-^ CD44^hi^ Tregs were sorted from both tissues and total ST2^-^ Tregs were sorted from spleen only. 100,000 splenic CD44^hi^ Tregs or 50,000 pulmonary CD44^hi^ Tregs were transferred into sublethally (450 rad) irradiated CD45.1 congenically marked hosts via tail vein injection. In some experiments, 1∗10^6^ total ST2^-^ Tregs from the spleen were transferred into sublethally (450 rad) irradiated CD45.1 congenically marked hosts via tail vein injection. Four weeks later, tissues were harvested for analysis. Splenocytes were enriched for CD4^+^ T cells using a CD4^+^ T cell isolation kit prior to staining and analysis.

#### Intravascular antibody labeling

Intravascular labeling of hematopoietic cells was performed based on previously published methods (Anderson et al., 2014). Three micrograms of PE-labeled anti-CD45.2 antibody was injected via the tail vein, and mice were euthanized with TBE three minutes after antibody injection. Tissues were then harvested for analysis. A pan-CD45 antibody was used for subsequent staining and identification of all CD45^+^ cells. Experiments were performed with and without perfusion; no differences were found in the fractions of i.v. labeled cells.

#### RNA sequencing and transcriptome analysis

For each Treg subset, 220 cells were sorted directly into reaction buffer using the SMART-Seq v4 Ultra Low Input RNA Kit for Sequencing (Takara), and reverse transcription was performed followed by PCR amplification to generate full length amplified cDNA. Sequencing libraries were constructed using the NexteraXT DNA sample preparation kit (Illumina) to generate Illumina-compatible barcoded libraries. Libraries were pooled and quantified using a Qubit® Fluorometer (Life Technologies). Dual-index, single-read sequencing of the pooled libraries was carried out on a HiSeq2500 sequencer (Illumina) with 58-base reads, using HiSeq v4 Cluster and SBS kits (Illumina) with a target depth of 5 million reads per sample. Reads were processed using workflows managed on the Galaxy platform. Base calls were processed to FASTQs on BaseSpace (Illumina), and a base call quality-trimming step was applied to remove low-confidence base calls from the ends of reads. Reads were trimmed by 1 base at the 3′ end then trimmed from both ends until base calls had a minimum quality score of at least 30. Any remaining adapter sequence was removed as well. The FASTQs were aligned to the GRCm38 mouse reference genome using STAR (v.2.4.2a) with the GRCh38 reference genome and gene annotations from ensembl release 91. Gene counts were generated using HTSeq-count (v0.4.1). Quality metrics were compiled from PICARD (v1.134), FASTQC (v0.11.3), Samtools (v1.2), and HTSeq-count (v0.4.1).

For quality control, samples that had counts >1 million, percent aligned >80% and median CV of gene coverage <0.7 were kept. Three libraries failed QC and were excluded from subsequent analyses. Counts were normalized using trimmed mean of M values (TMM) from the edgeR package (v3.26.8) (Robinson et al., 2010). Genes were filtered to include those that had a TMM normalization count of at least 1 count per millon in at least 10% of libraries and were classified as protein coding using BioMart (biomaRt (v2.40.5)). Only genes passing filter were included in downstream analyses. Gene counts were then transformed with observations level weights using voomWithQualityWeights from the limma R package (v3.40.6). Limma was then used to model the transcriptome expression changes within each group comparison. An adjusted p-value of 0.05 was used to determine statistical significance. All statistical tests were corrected by the Benjamini-Hochberg method. Principal Component Analysis was performed using the stats (base) R package, and function prcomp. Intraclass Correlation was performed using the ICC R package and function ICCBare. Other R packages used in the generation of heatmaps and plots include corrplot (v0.84), ComplexHeatmap (v2.2.0), and circlize (v0.4.12). Gene pathway enrichment analysis was performed using ShinyGO v0.74 (Ge et al., 2020). Hierarchical clustering analyses were performed using Euclidean distances and the complete linkage method except in Figure 4E, which used the Ward.D2 linkage method. The RNAseq datasets presented in this article have been submitted to the National Center for Biotechnology Information Gene Expression Omnibus (GEO: GSE182322).

### Quantification and statistical analysis

Statistical analyses of non-genomic data were performed using Prism (GraphPad Software). Data are displayed as individual data points with mean +/− standard deviation (SD). Comparison of the means was performed using unpaired, two-tailed Student’s t-tests or two-way ANOVA with Sidak’s multiple comparisons test, ns p > 0.05, ∗p < 0.05, ∗∗p < 0.01, ∗∗∗p < 0.001, ∗∗∗∗p < 0.0001. No statistical methods were used to predetermine sample size, but our sample sizes were similar to those generally employed in the field. No method of randomization was used.

## Data Availability

• RNAseq data have been deposited at GEO and are publicly available as of the date of publication. The accession number is listed in the key resources table. • This paper does not report original code. The codes used for RNAseq analysis followed typical pipelines from public R packages. • Any additional information required to reanalyze the data reported in this paper is available from the lead contact upon request.

## Supplemental information

Document S1. Figures S1–S8

Table S1. ST2 Treg RNAseq Datasets, related to Figure 4
