# Supplementary material for: Profiling of Tregs across tissues reveals plasticity in ST2 expression and hierarchies in tissue-specific phenotypes
Source: iScience. 2022 Aug 24;25(9):104998. doi: 10.1016/j.isci.2022.104998 (PMC9460833; doi:10.1016/j.isci.2022.104998)
Supplement: Document S1. Figures S1–S8 [file mmc1.pdf]

iScience, Volume *25*

Supplemental information

Profiling of Tregs across tissues reveals plasticity in ST2 expression and hierarchies in tissue-specific phenotypes

Sabine Spath, Florence Roan, Scott R. Presnell, Barbara Höllbacher, and Steven F. Ziegler

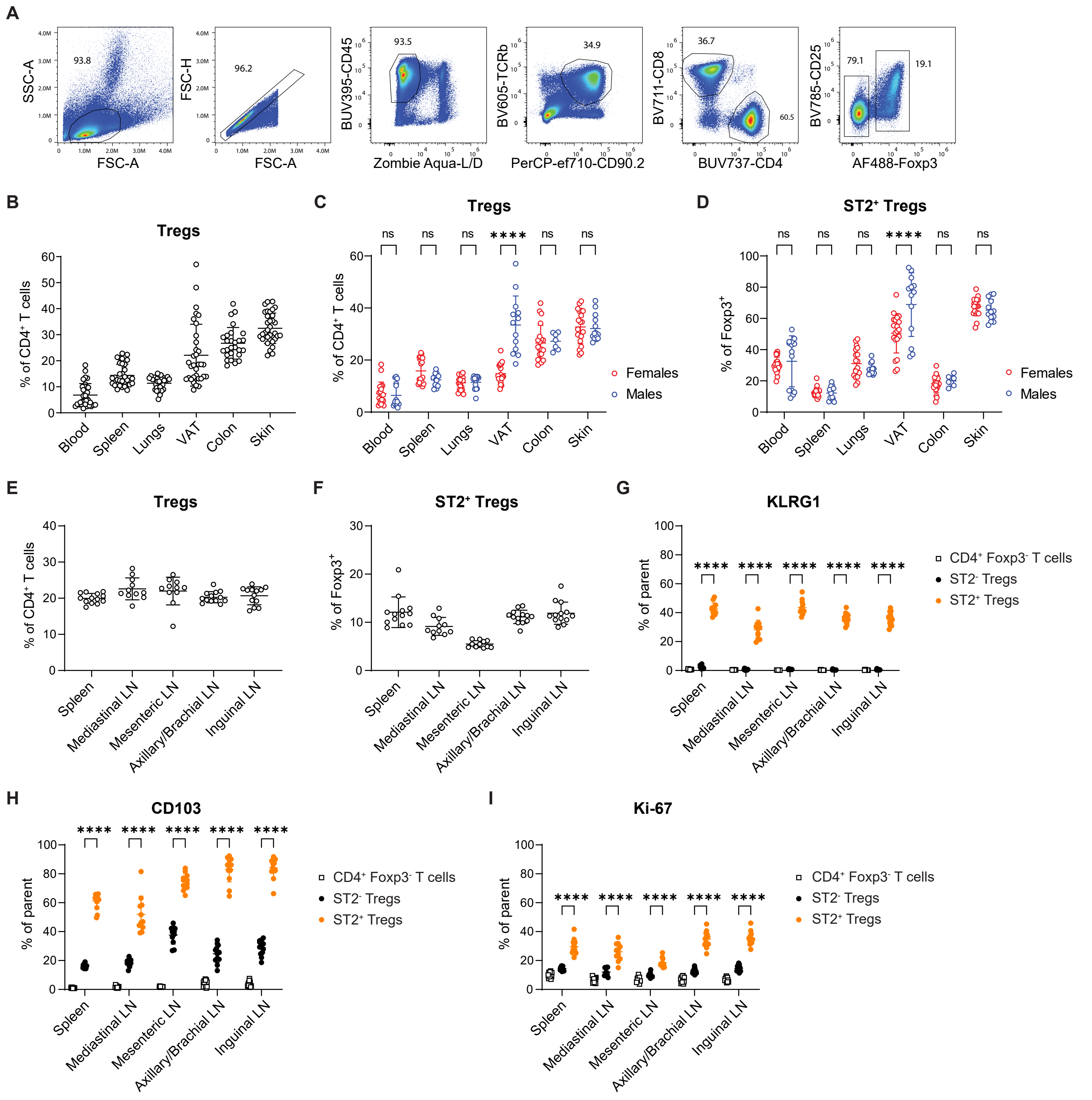

Figure S1. Flow cytometric analysis of Tregs across tissues and in secondary lymphoid organs, related to Figure 1. (**A**) Representative gating of splenic Tregs used in flow cytometric experiments. (**B**) Frequency of Foxp3$^+$ Tregs among all CD4$^+$ T cells across tissues. Data are pooled from 7 experiments, each with n = 4-6 per cohort. (**C**) Frequency of Foxp3$^+$ Tregs in females and males across tissues, based on Figure S1B. (**D**) Frequency of ST2$^+$ Tregs in females and males across tissues, based on Figure 1B. (**E and F**) Lymphocytes were pregated on live singlets, CD45$^+$, TCRβ$^+$ CD4$^+$ FOXP3$^+$ cells in spleen, mediastinal, mesenteric, axillary/brachial, and inguinal lymph nodes (LN). Frequency of (E) Foxp3$^+$ Tregs among all CD4$^+$ T cells and (F) ST2-expressing Tregs across secondary lymphoid organs. (**G-I**) Frequency of (G) KLRG1, (H) CD103, and (I) Ki-67 on ST2$^+$ Tregs, ST2$^-$ Tregs, and Foxp3$^-$ CD4$^+$ T cells across secondary lymphoid organs. E-I: Data are pooled from 2 experiments of n = 5 and n = 8. C+D, G-I: Indicated means were compared with 2-way ANOVA with Sidak's multiple comparisons test, ns $p > 0.05$, **** $p < 0.0001$. Data are plotted as mean +/- SD. Abbreviations: VAT: visceral adipose tissue, TCR: T cell receptor

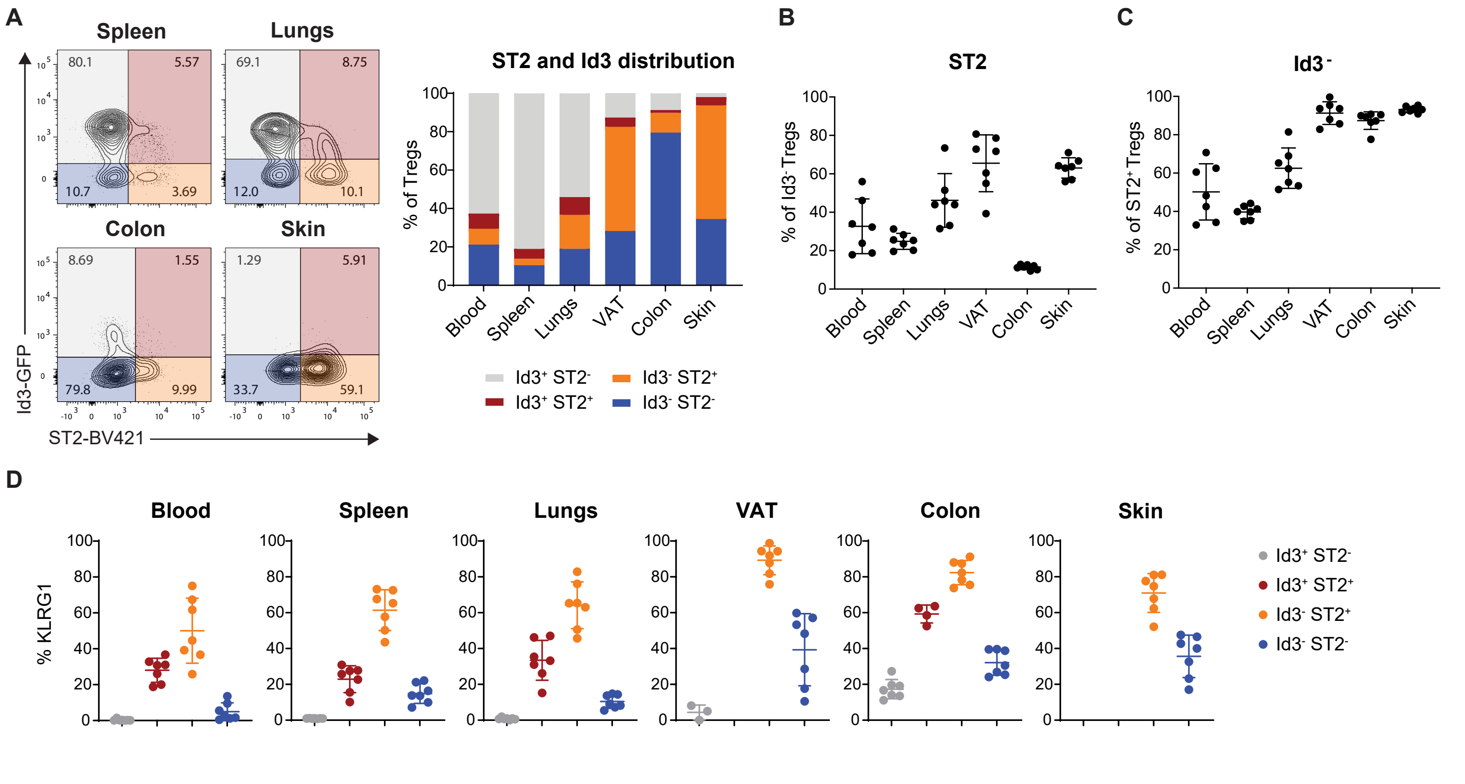

Figure S2. Correlation between Id3$^-$ Tregs and ST2$^+$ Tregs across tissues, related to Figure 2. (**A**) Relative distribution of Id3 and ST2 on Foxp3$^+$ Tregs in indicated tissues as assessed by flow cytometry using Foxp3^{mRFP}Id3^{GFP} reporter mice, left: representative flow plots, right: quantification of data pooled from two independent experiments with n = 3 and n = 4. (**B**) Frequency of ST2$^+$ cells among Id3$^-$ Tregs in indicated tissues. (**C**) Frequency of Id3$^-$ cells among ST2$^+$ Tregs in indicated tissues. (**D**) Frequency of KLRG1 on indicated Treg populations from Fig. S1A in blood, spleen, lungs, VAT, colon, and skin. Data points were removed for populations that routinely had less than 100 cells. B-D: Data are plotted as mean +/- SD. Abbreviations: VAT: visceral adipose tissue

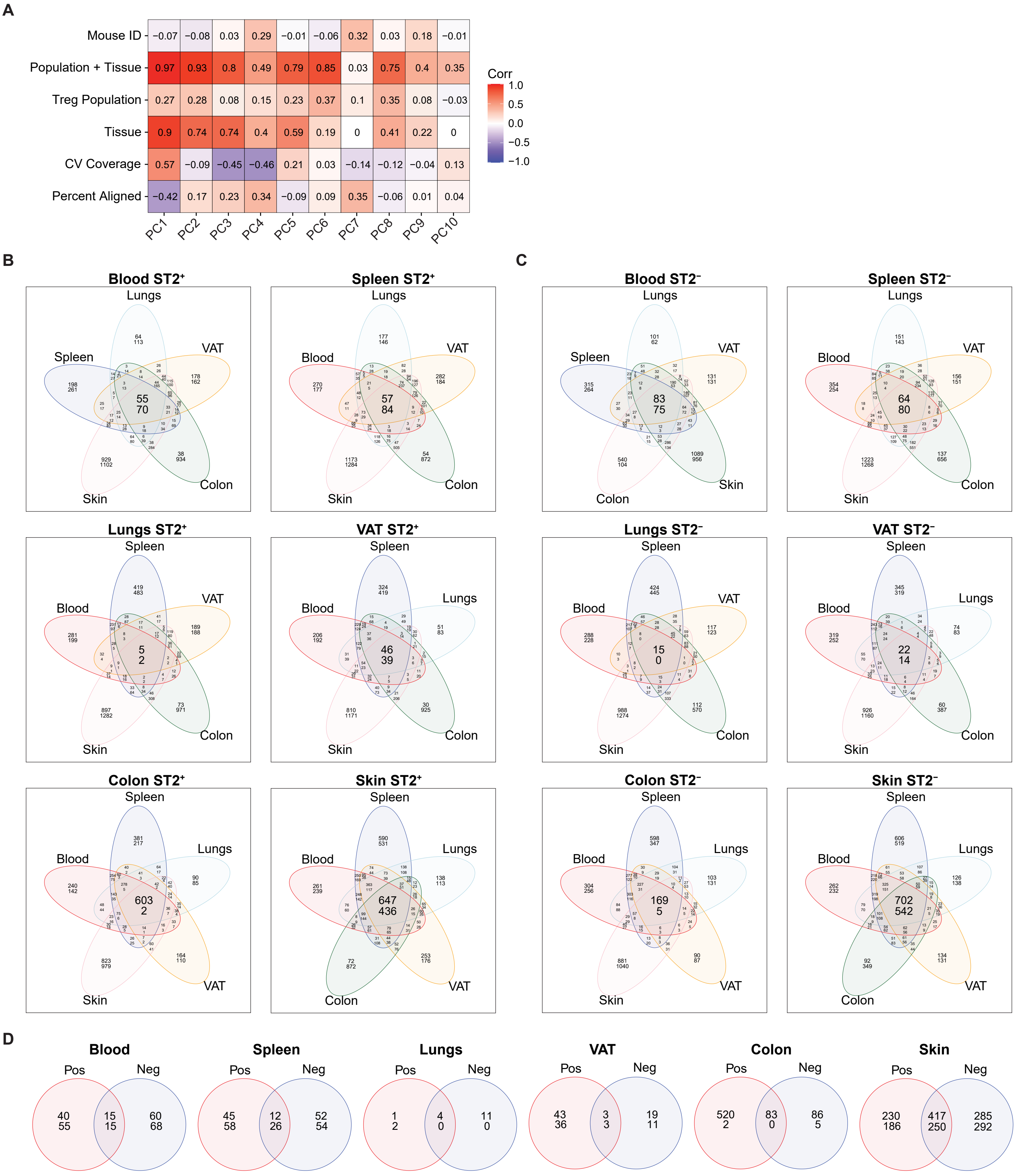
A
Mouse ID -0.07 -0.08 0.03 0.29 -0.01 -0.06 0.32 0.03 0.18 -0.01
Population + Tissue 0.97 0.93 0.8 0.49 0.79 0.85 0.03 0.75 0.4 0.35
Treg Population 0.27 0.28 0.08 0.15 0.23 0.37 0.1 0.35 0.08 -0.03
Tissue 0.9 0.74 0.74 0.4 0.59 0.19 0 0.41 0.22 0
CV Coverage 0.57 -0.09 -0.45 -0.46 0.21 0.03 -0.14 -0.12 -0.04 0.13
Percent Aligned -0.42 0.17 0.23 0.34 -0.09 0.09 0.35 -0.06 0.01 0.04
PC1 PC2 PC3 PC4 PC5 PC6 PC7 PC8 PC9 PC10
Corr
1.0
0.5
0.0
-0.5
-1.0
B
Blood ST2+
Spleen ST2+
Lungs ST2+
VAT ST2+
Colon ST2+
Skin ST2+
C
Blood ST2−
Spleen ST2−
Lungs ST2−
VAT ST2−
Colon ST2−
Skin ST2−
Blood
Spleen
Lungs
VAT
Colon
Skin
D
Blood
Pos Neg
40 55
15 15
60 68
Spleen
Pos Neg
45 58
12 26
52 54
Lungs
Pos Neg
1 2
4 0
11 0
VAT
Pos Neg
43 36
3 3
19 11
Colon
Pos Neg
520 2
83 0
86 5
Skin
Pos Neg
230 186
417 250
285 292

Figure S3. Correlation of factors/categorical variables across PC coordinates and Venn diagrams identifying tissue-specific DEGs, related to Figure 3. (**A**) Spearman's correlation for numeric variables and intraclass correlation coefficients for factor/categorical variables across coordinates of the PC plot. (**B and C**) Venn diagrams with tissue-specific DEGs identified in the central overlap for (B) $ST2^{+}$ $CD44^{hi}$ Tregs and (C) $ST2^{-}$ $CD44^{hi}$ Tregs by overlaying DEGs in one tissue versus each of the other tissues, repeated for each tissue. Genes with FC > 1.5 and adjusted p value < 0.05 were considered significant. (**D**) Venn diagram overlaying tissue-unique DEGs from $ST2^{+}$ and $ST2^{-}$ Tregs (identified from the central overlap of Venn diagrams in B and C) to identify DEGs in Tregs from the indicated tissue versus all other tissues. B-D: Upper numbers are upregulated genes; lower numbers are downregulated genes. Abbreviations: CV Coverage: median coefficient of variation of gene sequence coverage for the 1000 most highly expressed transcripts, Percent Aligned: percent aligned sequences to reference genome, VAT: visceral adipose tissue, DEG: differentially expressed genes

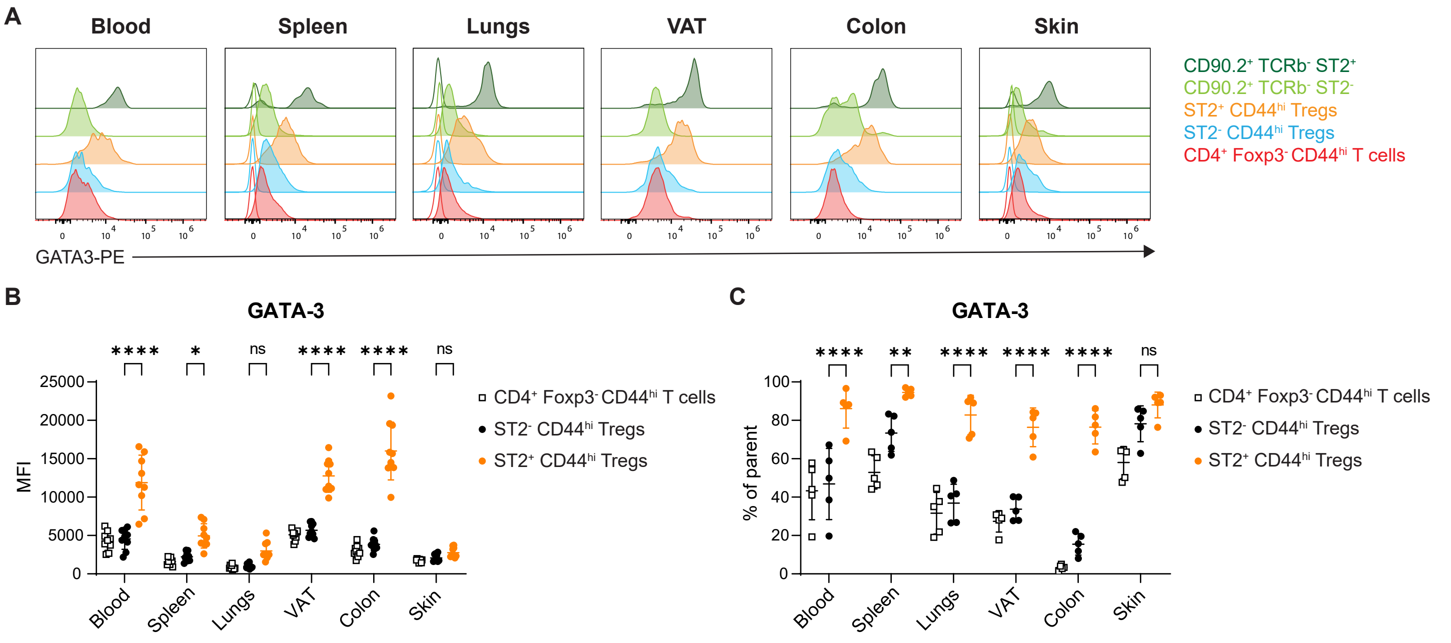

Figure S4. GATA3 flow cytometric analysis of ST2^{+} and ST2^{-} Tregs across tissues, related to Figure 4. (**A**) Representative, overlayed histograms showing GATA3 staining as assessed by flow cytometry in indicated cell populations and tissues, filled: full stain, open: FMO. T cell populations were pregated on live singlets, CD45^{+}, TCRβ^{+} CD90.2^{+} CD44^{hi} CD4^{+} cells; CD90.2^{+} TCRβ^{-} cell populations were pregated on live singlets, CD45^{+} cells. (**B**) Median fluorescence intensity (MFI) and (**C**) relative frequency of GATA3 on ST2^{+} CD44^{hi} Tregs, ST2^{-} CD44^{hi} Tregs, and Foxp3^{-} CD4^{+} CD44^{hi} T cells across tissues. B: Data are pooled from 2 experiments of n = 5 each. C: Data are from 1 experiment of n = 5. B and C: Data are plotted as mean +/- SD and indicated means were compared with 2-way ANOVA with Sidak's multiple comparisons test, ns $p > 0.05$, * $p < 0.05$, ** $p < 0.01$, **** $p < 0.0001$. Abbreviations: VAT: visceral adipose tissue, FMO: fluorescence minus one control, TCR: T cell receptor

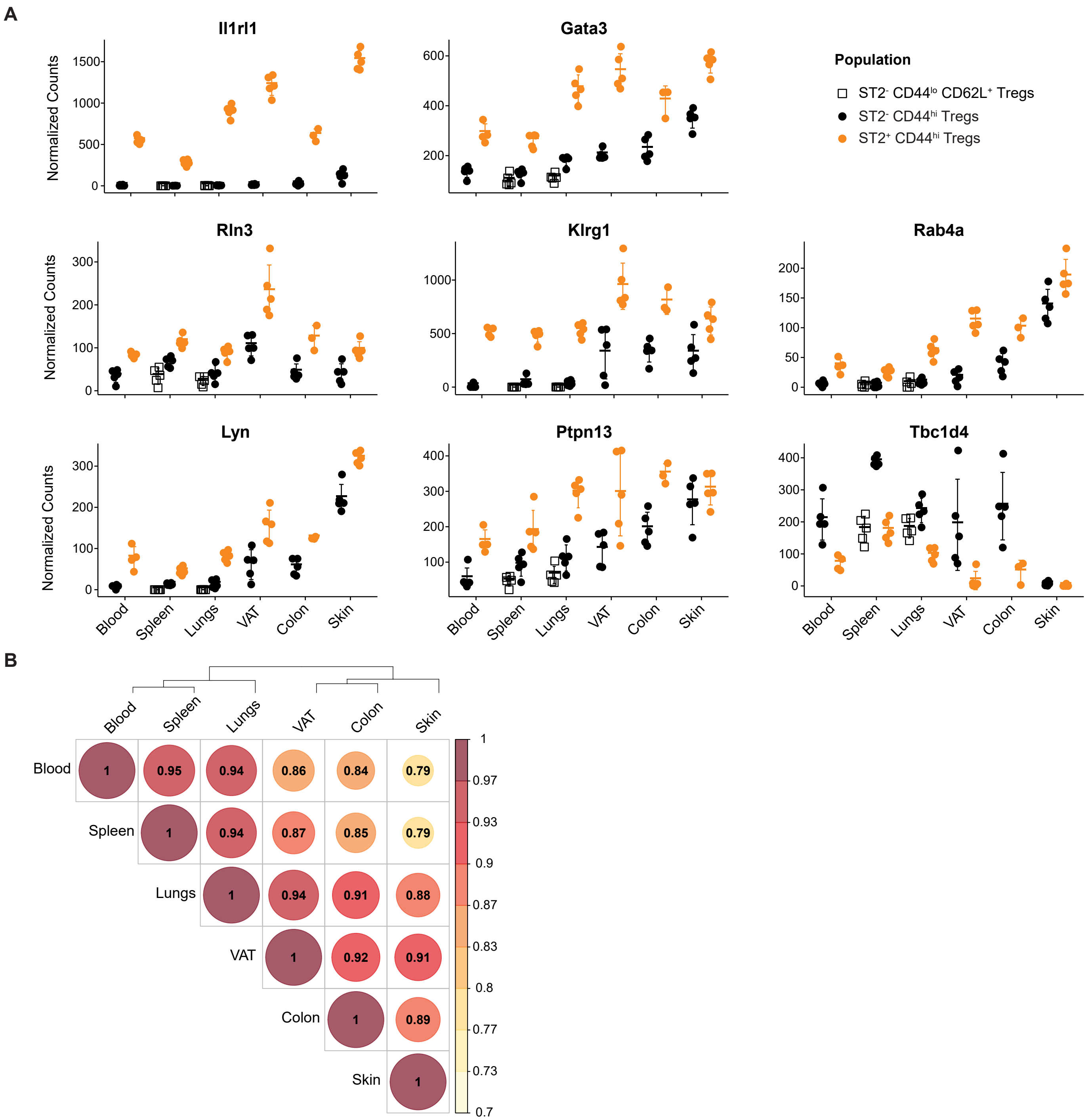

Figure S5. Expression of core DEGs between ST2$^+$ and ST2$^-$ Tregs across tissues, related to Figure 4. (**A**) RNAseq gene expression data, displayed as TMM normalized counts for DEGs between ST2$^+$ versus ST2$^-$ Tregs that are common to at least 5 of 6 tissues sampled, compare to Figure 4D. Data are plotted as mean +/- SD. (**B**) Dendrogram and correlation coefficient using Spearman's ranked-based correlation analysis of DEGs between ST2$^+$ versus ST2$^-$ Tregs pooled from all tissues. Abbreviations: VAT: visceral adipose tissue, DEG: differentially expressed genes

A

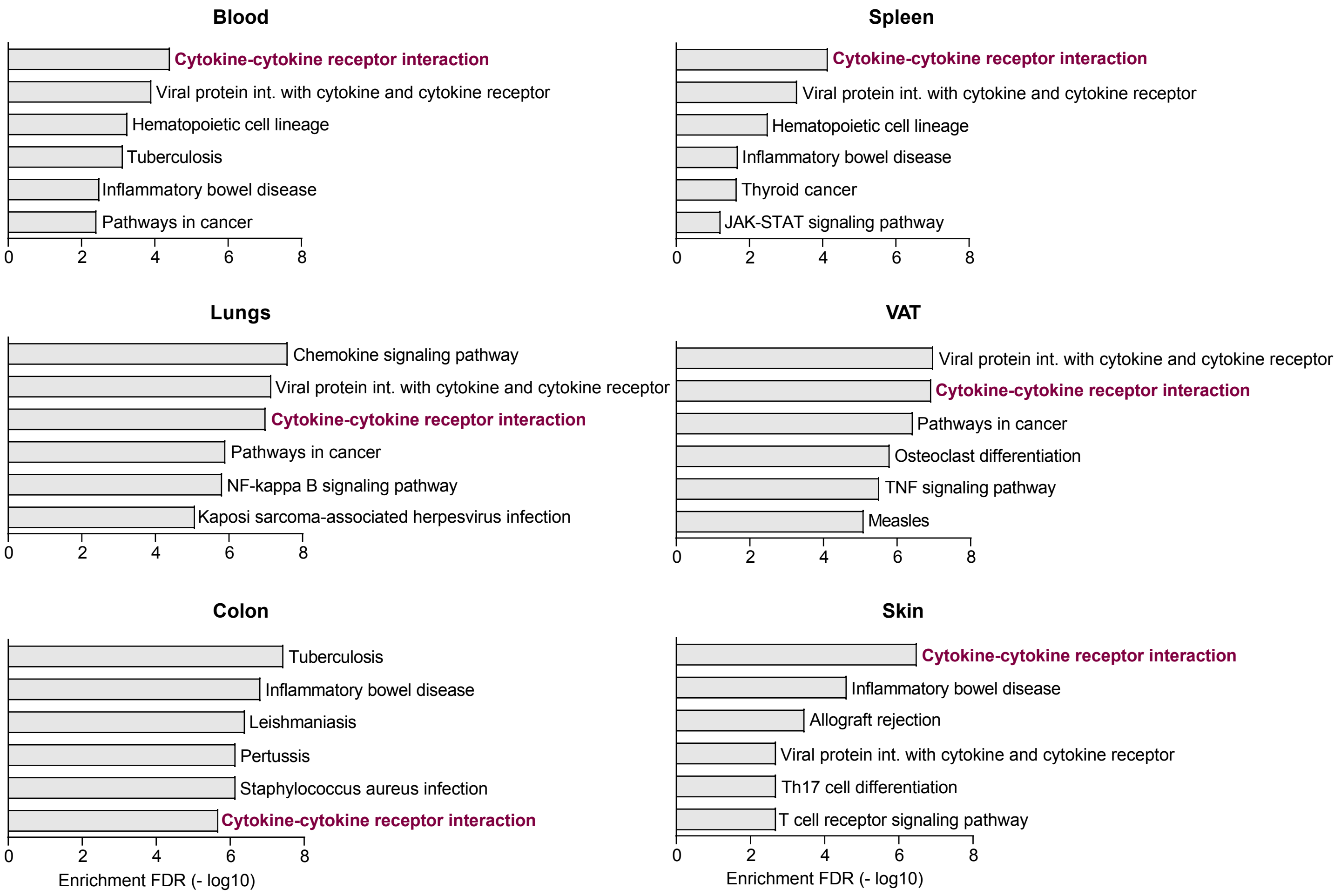

B

KEGG pathway: Cytokine-cytokine receptor interaction

Blood	CXCR5 BMPR1A CCR3 CCR2 CCR4 CCR7 CCR10 FAS IFNGR2 IL18 IL18RAP IL2RA IL6RA IL6ST IL7R IL9R IL1RL1 ACVR1C EBI3 TNFSF14 CLCF1 CXCR6 TNFRSF25
Spleen	CXCR5 CCR2 CCR4 EPOR IL10RA IL18RAP IL18R1 IL6ST IL7R IL9R LTA IL1RL1 CD40 EBI3 CXCR6
Lungs	CXCR5 CXCR3 CCR1 CCR3 CCR2 CCR4 CCR7 CCR8 CCR10 IFNGR2 IL10RA IL18RAP IL4 IL6RA IL9R IL1RL1 CXCL2 TNFRSF11A TNFRSF9 CXCL3 EBI3 IL17RB CXCR6
VAT	CXCR5 BMPR1A CXCR3 CCR1 CCR2 CSF1 CSF1R CSF2 FASL IFNG IL10 IL10RA IL18R1 IL2RA IL2RB IL5 IL7R IL9R IL1RL1 CCL5 CXCL2 TNFRSF11A TNFRSF14 CXCL3 TNFRSF25
Colon	BMP4 CCR6 CSF1R CSF2 CX3CR1 CXCL1 IL10RA IL1A IL1B IL1RL1 CCL11 CCL20 CCL3 CCL6 IL23R TNFSF11 ACKR4 CXCL16 IL33
Skin	CXCR3 FAS IFNG IL18RAP IL5 IL1RL1 CCL8 CXCL2 CD40LG TNFRSF25

Figure S6. Enriched KEGG pathways in DEGs between ST2$^+$ and ST2$^-$ Tregs in each tissue, related to Figure 4. (**A**) KEGG pathways enriched in DEGs between ST2$^+$ and ST2$^-$ Tregs in each tissue were determined using ShinyGO (Ge et al., 2020). Barplots show the top six most significantly enriched terms in each tissue. (**B**) Gene list of DEGs that are part of the KEGG pathway: Cytokine-cytokine receptor interaction for each tissue. Abbreviations: int: interaction, DEG: differentially expressed genes, KEGG: Kyoto Encyclopedia of Genes and Genomes Database

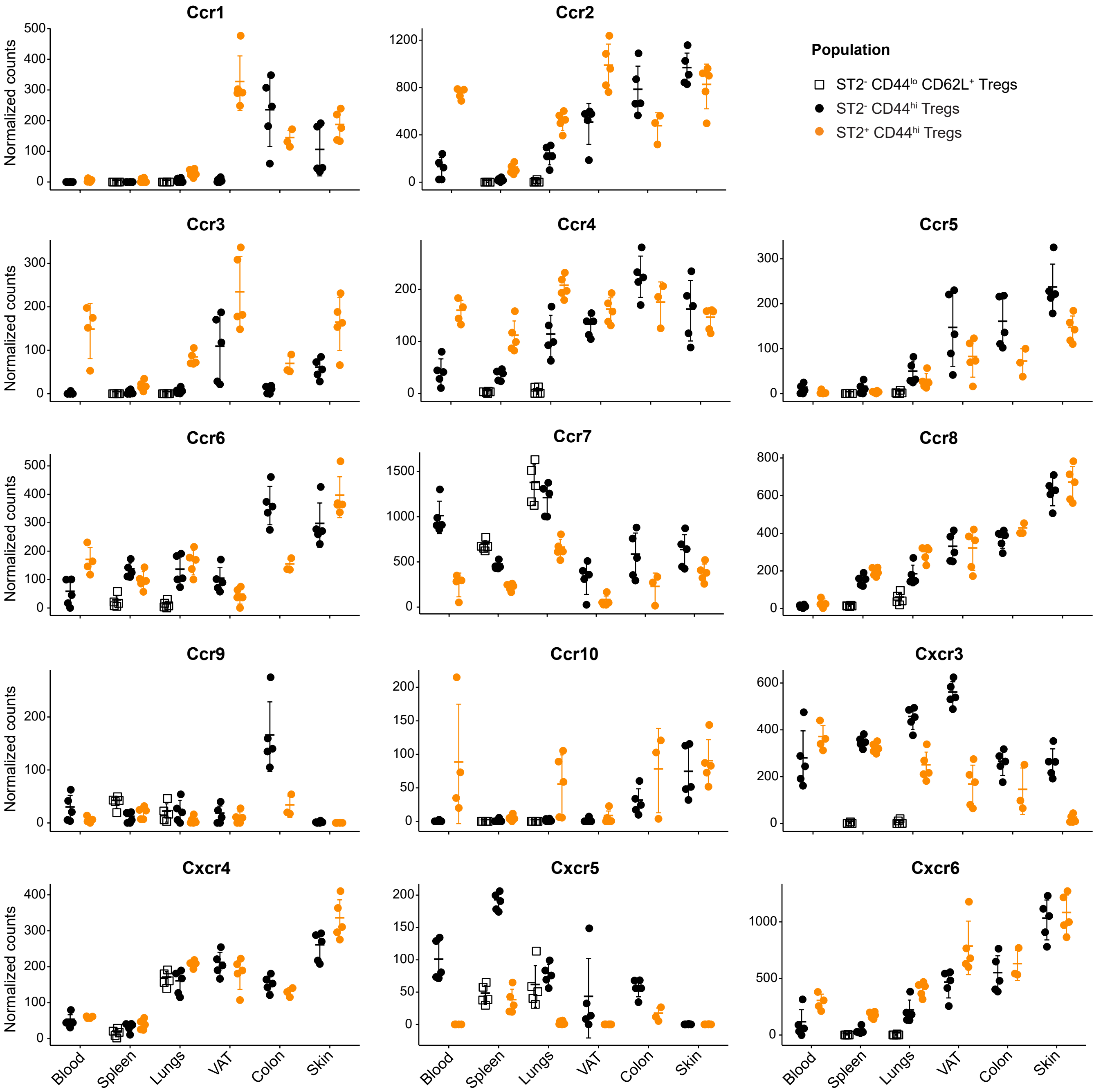

Figure S7. Chemokine receptor gene expression profiles of ST2^{+} and ST2^{-} Tregs across tissues, related to Figure 5. RNAseq gene expression data displayed as TMM normalized counts for indicated chemokine receptors. Data are plotted as mean +/- SD. Abbreviations: VAT: visceral adipose tissue, CCR: CC chemokine receptor, CXCR: CXC chemokine receptor

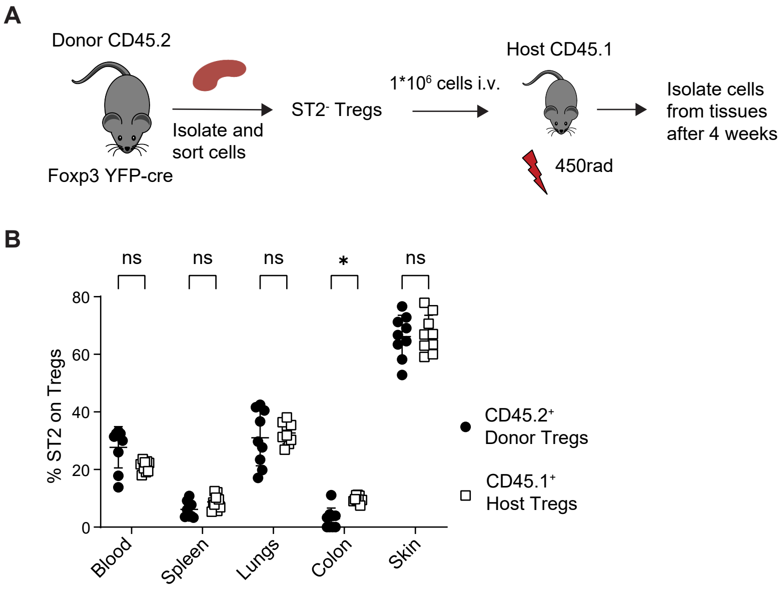

Figure S8. Adoptive transfer of $1*10^6$ total ST2$^-$ Tregs from the spleen into sublethally irradiated WT hosts, related to Figure 6. (**A**) Experimental setup: ST2$^-$ Tregs were sorted from the spleen of CD45.2 Foxp3$^{YFP\text{-}cre}$ mice and transferred i.v. into sublethally irradiated CD45.1 hosts. Organs of host mice were harvested 4 weeks after transfer to assess recovery of transferred Tregs. (**B**) ST2 expression on recovered donor CD45.2$^+$ Tregs and host CD45.1$^+$ Tregs in the indicated tissues after transfer. Lymphocytes were gated on live singlets, CD4$^+$ FOXP3$^+$ cells. Data are plotted as mean +/- SD and pooled from 2 experiments of n = 3 and n = 6. Indicated means were compared with 2-way ANOVA with Sidak's multiple comparisons test, ns $p > 0.05$, * $p < 0.05$. Abbreviations: VAT: visceral adipose tissue
